# Radical Polyesters: Connecting Spacer Structure to Bulk Electrical Conductivity

**DOI:** 10.1021/acsmacrolett.6c00245

**Published:** 2026-06-18

**Authors:** Kieran G. Stakem, Simon J. Cassidy, William K. Myers, Georgina L. Gregory

**Affiliations:** † Department of Chemistry, 6396 University of Oxford, 12 Mansfield Road, Oxford OX1 3TA, United Kingdom; ‡ Department of Chemistry, University of Oxford, South Parks Road, Oxford OX1 3QR, United Kingdom; § Centre for Advanced Electron Spin Resonance (CAESR), Department of Chemistry, University of Oxford, South Parks Road, Oxford OX1 3QR, United Kingdom

## Abstract

Electron exchange communication between nitroxide radical sites localized along polymer backbones creates a compelling platform for spin electronics, resistive memory, and optoelectronics. While radical site proximity, chain flexibility, and local ordering form the basis for this communication, how site-to-site spacer structure governs bulk redox charge transfer remains an open question. Herein, epoxide-cyclic anhydride ring-opening copolymerization produces TEMPO-functional radical polyesters, where strictly alternating enchainment installs a radical at every repeat unit while anhydride comonomer varies spacer structure from flexible aliphatic through alicyclic, bicyclic, and semiaromatic. SQUID magnetometry and EPR spectroscopy confirm radical contents of 86–98%; except for the thioether-containing polyester, where sulfur-specific quenching occurs. Density functional theory calculations reveal that rigid aromatic spacers position radical sites closer than flexible aliphatic ones of comparable through-bond atom counts. However, solid-state electrical conductivity measurements demonstrate that glass transition is the primary determinant of bulk charge transport, regardless of whether it is set through spacer flexibility, blending, or block copolymerization.

## Graphical abstract

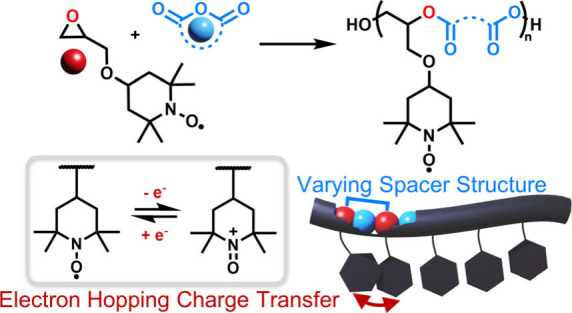

Nitroxide radical polymers are an emerging class of electronic material. Unlike doped π-conjugated polymers, electronic activity arises from fast, reversible redox exchange between pendant air-stable radical sites. This redox charge transfer enables metal-free electronic function in processable, optically transparent materials.^1,2^ Accordingly, radical polymers have been explored in a range of applications including energy storage,^3–7^ rewritable memory devices,^8,9^ thermoelectrics,^10^ electrochromic displays,^11,12^ solar cells,^13,14^ spintronics,^15,16^ and medical contrast agents,^17^ with growing relevance to solid-state flexible electronics.^18^ Among nitroxide radicals, (2,2,6,6-tetramethylpiperidin-1-yl)­oxyl (TEMPO) has attracted the most attention due to its air stability, synthetic accessibility, and reliable one-electron redox chemistry. Various TEMPO-based polymers have consequently been investigated for electronic applications, from early TEMPO-functionalized polymethacrylate (PTMA)^19^ to the development of polyether-backboned PTEO.^20–22^ Despite this breadth of interest, charge transport performance remains limited, and establishing clear structure–property relationships is essential to guide further development.

Electronic communication in TEMPO-functional polymers is generally understood to proceed via two principal mechanisms: self-exchange electron hopping between nearby radical centers, described within the Marcus–Hush framework, and a diffusion-cooperative mechanism in which pendant-group Brownian motion brings localized radical sites into proximity for electron transfer.^23–25^ High solid-state electrical conductivity was first demonstrated with PTEO, synthesized by anionic ring-opening polymerization of GTEMPO epoxide (4-glycidyloxy-2,2,6,6-tetramethylpiperidine-1-oxyl). Its flexible ether backbone and low glass transition temperature (*T*_g_) facilitate percolating radical networks, enabling conductivities of ca. 0.2 S cm^–1^, yet only at channel lengths ≤600 nm.^20^ Replacement of the backbone oxygen with sulfur alters chain conformation and the electronic distribution around the radical site, extending measurable conductivity to the 1.5 μm scale.^26^ Beyond backbone heteroatom, stereochemical regularity enhances conductivity by several orders of magnitude in radical polymonothiocarbonates,^27^ and backbone spacer length, modulated through carbon linker, has been correlated with *T*_g_ and identified as a key factor in redox kinetics.^28^ Computational mapping of backbone, radical, and spacer combinations has begun to illuminate these relationships systematically.^29^ Sustainability has also motivated TEMPO-functional polymers bearing biodegradable^30,31^ and bioderived backbones^32^ with relevance to transient and biointegrated electronics. However, across this body of work, the effect of backbone site-to-site spacer flexibility on solid-state charge transport has not been reported.

In this paper, we exploit the backbone diversity accessible through epoxide-cyclic anhydride ring-opening copolymerization (ROCOP) to prepare a series of TEMPO-functional radical polyesters with systematically varied backbone spacer structures. ROCOP is an established route to well-defined alternating polyesters, offering control over the molecular weight, dispersity, and backbone composition through monomer selection. Pioneered with metal-based catalysts^33–35^ and extended to organocatalytic systems,^36,37^ ROCOP provides access to functional polyesters from readily available, often biobased monomers,^38–40^ with the resulting backbones being inherently degradable.^41^ This chemistry has been applied to GTEMPO via carbonyl sulfide copolymerization, yielding isotactic and atactic radical polymonothiocarbonates.^27^ Here, we combine synthesis, thermal analysis, magnetometry, and computation to establish how backbone spacer structure between TEMPO-radical sites, varied from flexible aliphatic to rigid semiaromatic, governs bulk solid-state electrical conductivity (charge transport averaged across local pathways through thick films, where interfacial contributions are minimized).

TEMPO-functionalized radical polyesters were synthesized by ROCOP of GTEMPO with a series of cyclic anhydrides, catalyzed by the phosphazene organobase *t*-Bu-P_2_ in 2-MeTHF at 60–75 °C (Scheme 1, see SI for details). GTEMPO is accessible from bioderived epichlorohydrin.^42^ Anhydride comonomers were selected to modulate spacer structure and, where possible, exploit renewable feedstocks: glutaric anhydride (GA), diglycolic anhydride (DGA), and thiodiglycolic anhydride (TDGA) provide flexible spacers incorporating heteroatoms O and S; phthalic anhydride (PA) and 4-methylphthalic anhydride (MPA) provide semiaromatic spacers; hexahydrophthalic anhydride (HHPA) and tricyclic anhydride (TCA) provide alicyclic and sterically encumbered bicyclic spacers, respectively. The alternating enchainment of ROCOP, combined with the TEMPO side chain steric bulk enforcing head-to-tail selectivity, installs TEMPO at every repeat unit with regular spacing along the backbone. A chain transfer agent (1,4-benzene dimethanol, BDM) was employed to target low degrees of polymerization (DP < 10), affording number-average molecular weights (*M*_n_) of 2000–4000 g mol^–1^ and dispersities (*Đ*) below 1.4 by size-exclusion chromatography (SEC) relative to narrow polystyrene standards (Figure S2); TDGA was an exception (*Đ* = 1.8), attributed to retarded propagation through sulfur coordination to the P_2_ catalyst. Low DP gave comparable, subentanglement *M*_n_ across the series, since entanglement-constrained chain dynamics might otherwise confound the spacer-structure comparison. MALDI-ToF mass spectrometry confirmed BDM incorporation, the correct alternating repeat units and hydroxyl chain ends (Figures S3 and S4). Paramagnetic TEMPO radicals were reduced to the hydroxylamine using pentafluorophenylhydrazine prior to ^1^H and ^13^C NMR (Figures S5–S13). Degradability of the polyesters was confirmed by subjecting an example in the series to basic hydrolysis (1 M NaOH), which yielded the corresponding diacid and diol products (Figure S14).

**Scheme 1:**
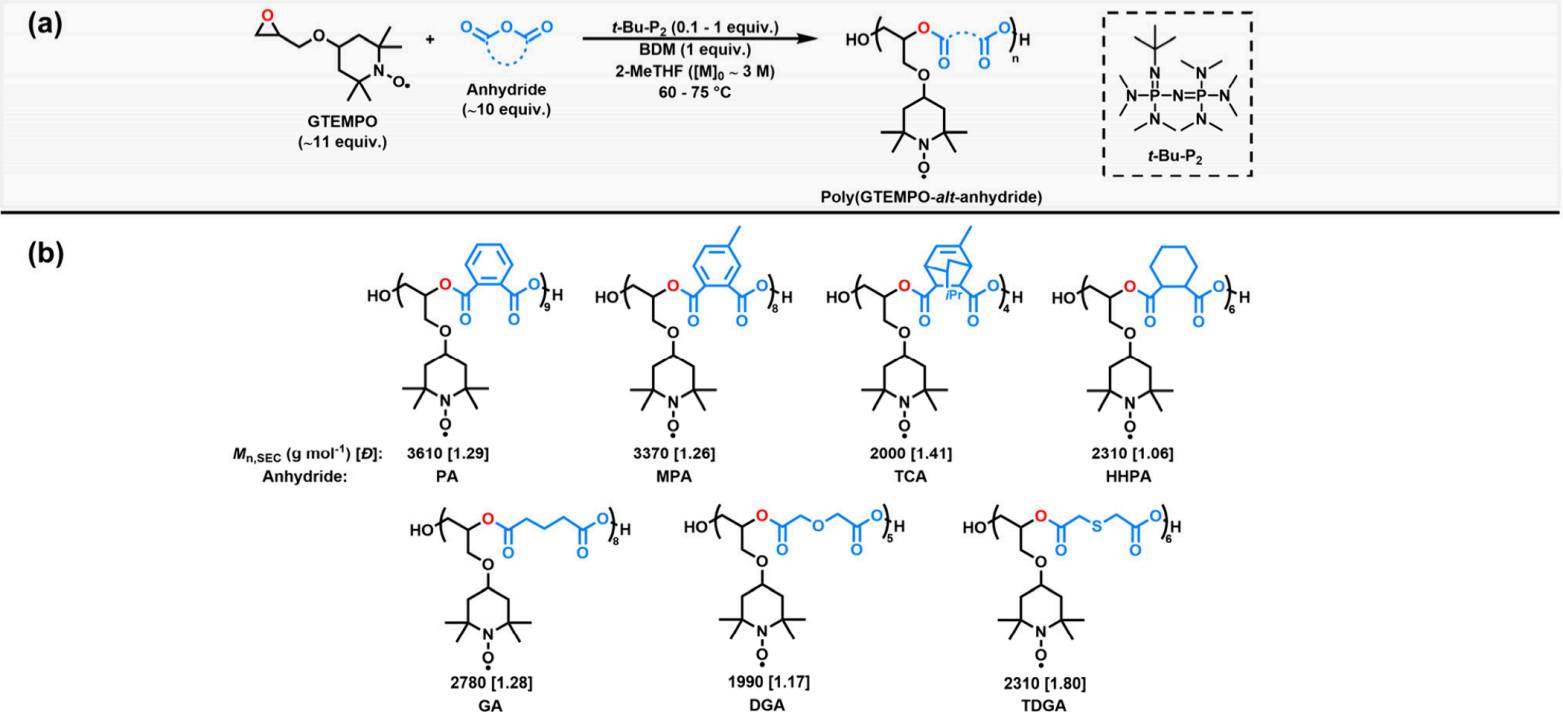
(a) Synthesis of Poly­(GTEMPO-*alt*-anhydride) Radical Polyesters by ROCOP*; (b) Chemical Structures and Apparent *M*_n_ (g mol^–1^), *Đ* (=*M*_w_/*M*_n_) by SEC^†^; *n* = (*M*_n,SEC_ – *M*_r,BDM_)/*M*_r,repeat unit_ ^a^

a *See Table S1 for anhydride-specific quantities and conditions; ^†^THF eluent, relative to PS standards; RI detector, UV for semi-aromatic polyesters.

The backbone spacer structure afforded by the anhydride comonomers was first interrogated computationally to understand how it translates into though-space radical–radical geometry. DFT calculations were performed on DP = 2 model oligomers, treating neutral open-shell diradical, singly oxidized TEMPO^+^/TEMPO, and doubly oxidized TEMPO^+^/TEMPO^+^ charge states (Figure 1, see SI). Assuming head-to-tail enchainment as established above, the through-bond atom count between adjacent TEMPO pendant points is 4 for poly­(GTEMPO) (PTEO), reflecting its compact ether backbone, while the ROCOP polyesters access a longer regime: 9-atom spacers for PA, MPA, TCA, and HHPA, and 10-atoms for GA, DGA, and TDGA. From DFT-optimized geometries, the flexible aliphatic spacers GA and DGA, and the thioether TDGA, give N···N distances of ca. 20 Å, consistent across spacer identity and oxidation state. In contrast, the rigid phthalic anhydride-derived spacers give shorter distances: the ortho-substituted geometry, with both ester linkages emerging from adjacent ring positions, enforces a sharp chain kink that curves the backbone back on itself, bringing TEMPO units closer. PA and TCA give N···N spacings of 7–8 Å, invariant across oxidation states, while HHPA and MPA give intermediate distances of ca. 10 Å, though with greater variation across oxidation states reflecting sensitivity of the N···N geometry to the singly oxidized configuration. Spin density analysis, SOMO energy levels, and natural bond orbital (NBO) charges confirm that TEMPO radical character is preserved across the series, with discrete, nonoverlapping radical sites throughout (Table S2). TDGA is the single electronic anomaly: NBO analysis reveals a positive charge on sulfur (+0.203 e, cf. −0.561 e and −0.382 e on the corresponding O and C positions in DGA and GA respectively, Figure S15), an electron-deficiency that may be relevant to the reduced radical content observed subsequently for this polymer.

**Figure 1:**
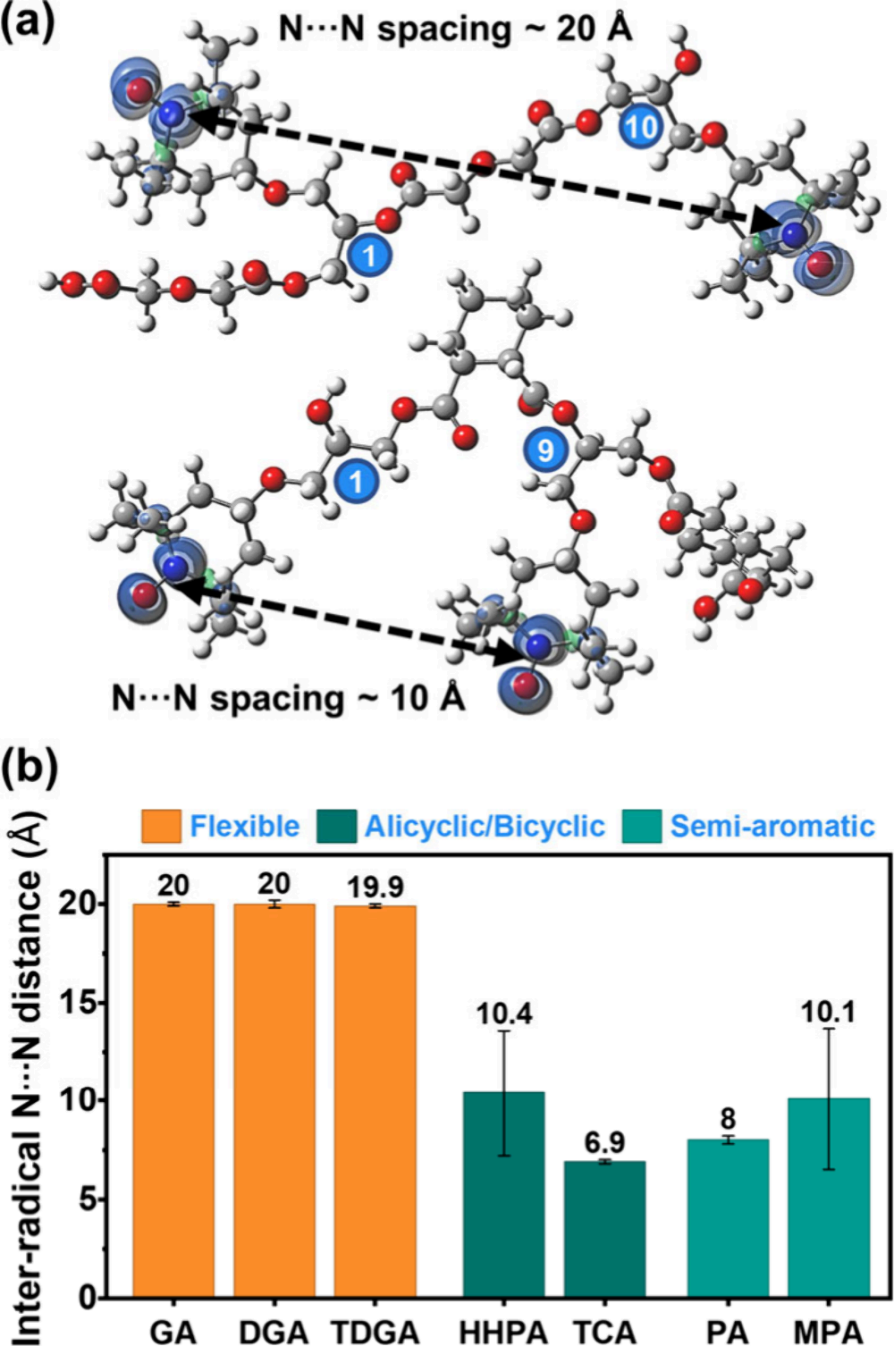
(a) N···N distance in DFT-optimized geometries (ωB97X-D/6–311G­(d,p)) for representative flexible aliphatic (top, DGA) and rigid (bottom, HHPA) spacers with spin density surfaces (isovalue = 0.002). End-to-end through-bond atom count in blue (10 and 9 atoms, respectively). (b) N···N spacings across anhydrides; bars show the mean over diradical, singly and doubly oxidized states; error bars, the standard deviation.

Beyond this geometry of radical sites in space, the backbone spacer structure governs their mobility and thus their propensity to move into proximity for electron transfer. Backbone chain mobility was probed by differential scanning calorimetry (DSC) and revealed that anhydride selection enables variation of *T*_g_ from below to well above room temperature (Figure 2a). The flexible aliphatic polyesters exhibited the lowest values: GA (17 °C), DGA (44 °C), and TDGA (58 °C), reflecting progressive stiffening of the backbone as heteroatom polarizability increases from O to S. The semiaromatic and alicyclic, bicyclic polyesters formed a clustered group at higher *T*_g_: HHPA (73 °C), PA (74 °C), TCA (75 °C), and MPA (79 °C), spanning only 6 °C despite their structural diversity, consistent with backbone rigidity as the dominant determinant of chain mobility in this subgroup. All polymers were fully amorphous, with no crystallization or melting transitions observed, attributed to the steric bulk of the TEMPO side chain inhibiting regular chain packing. Thermogravimetric analysis (TGA) confirmed good thermal stability across the series, with onset of 5% mass loss (*T*_d,5%_) between 210 and 259 °C, well above the temperatures used in conductivity measurements (Figures 2b and S17).

**Figure 2:**
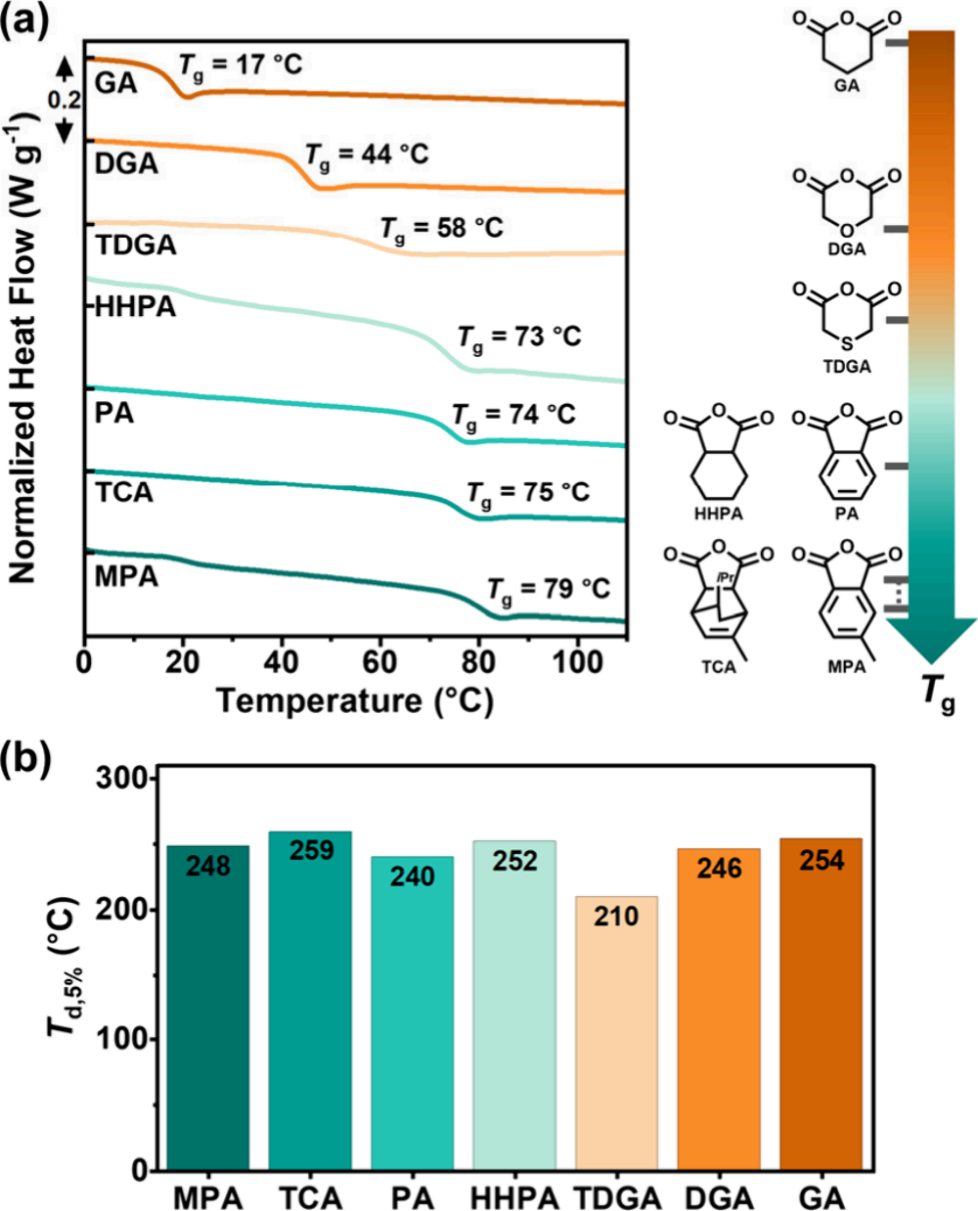
(a) DSC thermograms of P­(GTEMPO-*alt*-anhydrides) showing *T*_g_ (Exo up, 3rd heat cycle, 10 °C min^–1^). (b) Decomposition temperatures (*T*_d,5%_, 5% mass loss by TGA).

Next, the radical content and magnetic behavior of the polyester series were assessed by superconducting quantum interference (SQUID) magnetometry.^43^ Temperature-dependent magnetic susceptibility data were fitted to the Curie–Weiss law, from which radical contents of 86–98% per repeat unit across most of the series were determined (Figure 3a,b, S19). This confirmed that the ROCOP synthesis and polyester backbone do not compromise radical integrity. TDGA was an outlier, exhibiting a reduced radical content of ca. 54% per repeat unit. While thioether backbones can support high radical content,^26^ the reduction for TDGA is likely sulfur-associated, consistent with its anomalous polymerization behavior and the electron deficient sulfur, both noted above. Weiss constants (θ_CW_) of −0.51 ± 0.06 K confirmed weak antiferromagnetic exchange, indicating that radicals are essentially quasi-independent with only slight preference for antiparallel spin alignment, as expected for TEMPO sites separated by nonconjugated spacers. Electron paramagnetic resonance (EPR) spectroscopy in solution on representative samples (GA and PA) indicated high spin concentration, evident from the substantial collapse of the *A*(^14^N) hyperfine structure toward a Lorentzian line shape, with radical contents in agreement with SQUID magnetometry (Figure 3c).^44^ FTIR confirmed the N–O^•^ stretch in the region of 1370–1355 cm^–1^ alongside the characteristic polyester carbonyl (1721 cm^–1^), with no evidence of oxoammonium or hydroxylamine species (Figure S21). Finally, cyclic voltammetry (CV) confirmed the nitroxide/oxoammonium redox couple at ca. +0.34/+0.25 V vs Fc/Fc^+^, with peak separations of 90 mV at 0.1 V s^–1^, consistent with outer-sphere electron transfer (Figure 3d).

**Figure 3:**
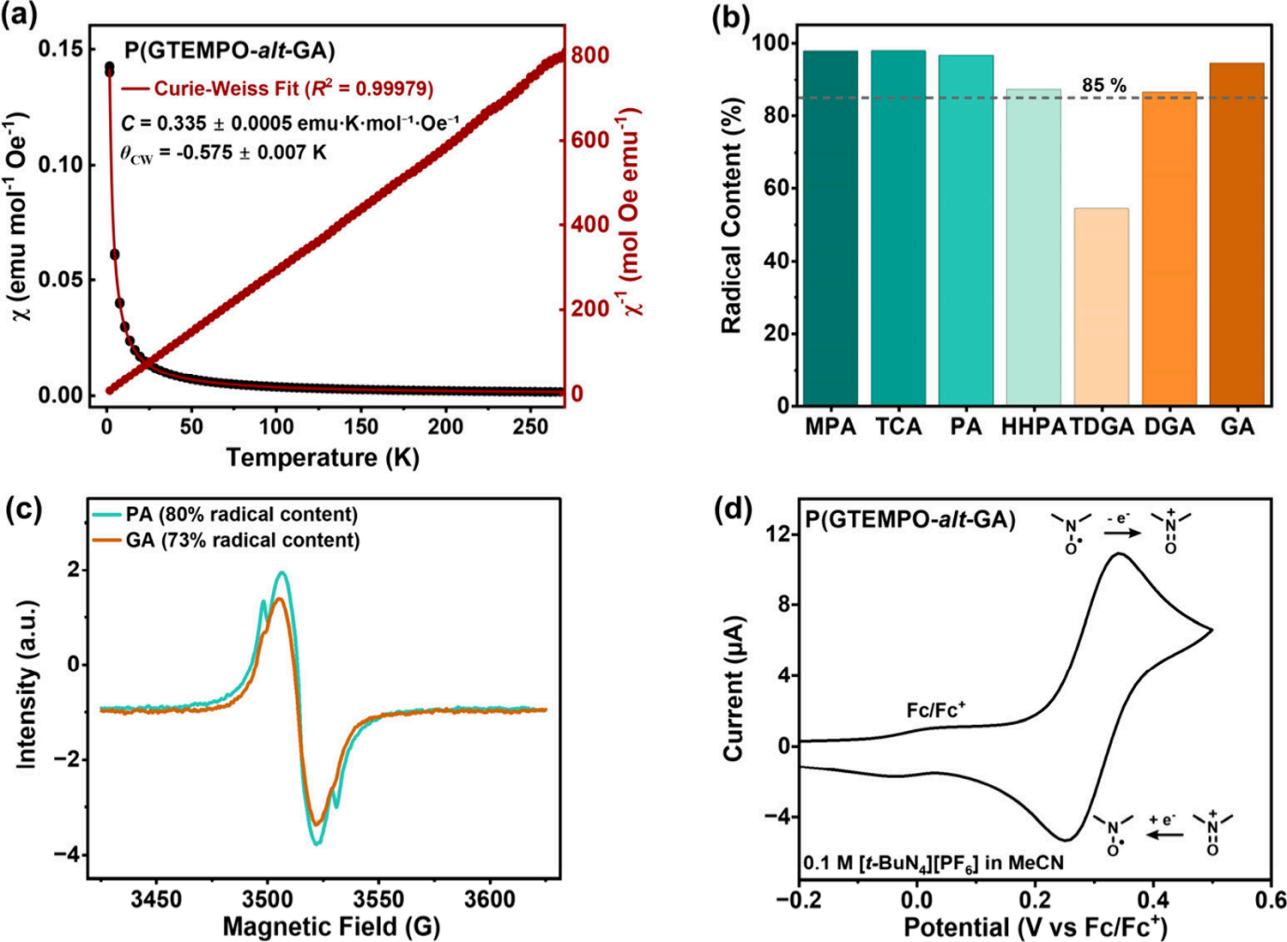
(a) Representative molar magnetic susceptibility (χ) and inverse susceptibility (1/χ) vs *T*; Curie–Weiss fit of χ vs *T* (nonlinear least-squares), with linear 1/χ vs *T* confirming Curie–Weiss behavior (see SI for χ*T* vs *T*). (b) Radical content for P­(GTEMPO-*alt*-anhydrides) by SQUID. (c) Solution EPR spectra for selected flexible and rigid spacers; radical content (parentheses) by double integration. (d) Characteristic solution CV (ca. 5 mM TEMPO) showing TEMPO/TEMPO^+^ couple, referenced to ferrocene (Fc/Fc^+^).

Having established comparable radical content across the series, solid-state electrical conductivities were measured by linear sweep voltammetry (LSV) using through-plane symmetric gold electrodes in a sandwich cell. Thick films (ca. 900 μm) provide a bulk-dominated response, supported by electrochemical impedance spectroscopy (Figure S23). Under applied bias, conduction proceeds by oxidation of neutral nitroxide to oxoammonium at the positively biased electrode, self-exchange between oxidized and neutral sites through the sample, and reduction back to nitroxide at the opposite electrode, with charge balance maintained through the external circuit. Ohmic behavior was confirmed from the linearity of the resulting current–voltage (*I*–*V*) curves with resistance (*R*) extracted from the slope and conductivity calculated as σ = *L*/*RA*, where *L* is film thickness and *A* is electrode contact area (Figure 4a). Samples were heated above their respective *T*_g_ to ensure good electrode contact, with measurements taken on cooling. The rigid aromatic, alicyclic, and bicyclic polyesters (PA, MPA, TCA, HHPA), measured at 100–120 °C, all exhibited conductivities of order 10^–9^ S cm^–1^ (Figure 4b). Within this series, HHPA and PA showed comparable conductivities consistent with their near-identical *T*_g_ values, while MPA and TCA were slightly lower. The narrow *T*_g_ range (73–79 °C) across this group is consistent with the similarly narrow range of conductivities observed, despite differences in N···N distance. These distances are likely too large to support efficient intrachain electron transfer, and conductivity is therefore primarily attributed to interchain hopping.

**Figure 4:**
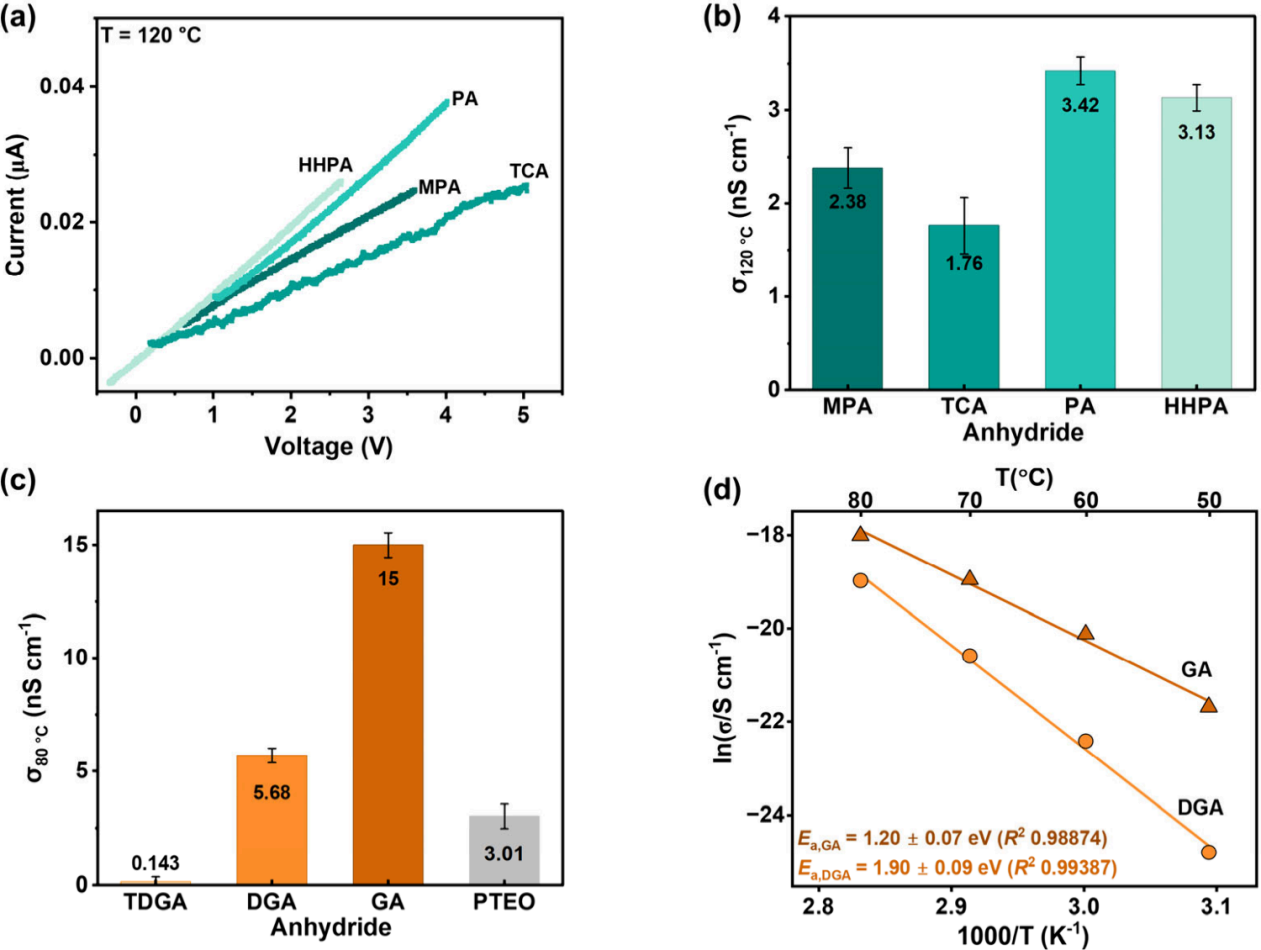
(a) Solid-state *I*–*V* curves (scan rate 1 mV s^–1^) for rigid spacers. (b, c) Electrical conductivities (σ) above *T*_g_ for rigid and flexible spacers, respectively; PTEO included as reference. (d) Arrhenius plots of σ and calculated activation energies (*E*_a_).

In contrast, the flexible polyesters spanned nearly 3 orders of magnitude in conductivity at 80 °C (Figure 4c), tracking their broader *T*_g_ range. GA exhibited the highest value of 1.5 × 10^–8^ S cm^–1^, approximately an order of magnitude above the rigid series, consistent with its lowest *T*_g_ and greatest chain mobility. For comparison, PTEO (*T*_g_ ∼ 22 °C), measured under identical conditions and at comparable film thickness, exhibited lower conductivity than P­(GTEMPO-*alt*-GA). Higher values reported in the literature for PTEO were obtained over nanometer channel lengths, where local radical ordering is thought to be sustained across the electrode gap, far below the present thick-film length scales. The low conductivity of P­(GTEMPO-*alt*-TDGA) is consistent with its reduced radical content and elevated *T*_g_ compared to the GA polyester. It contrasts with the thioether analogue of PTEO (PTES),^26^ where sulfur substitution enhanced conductivity, suggesting the effect of sulfur is context dependent and potentially sensitive to its main chain position.

Temperature-dependent conductivity followed Arrhenius behavior across the series, consistent with thermally activated charge transport where enhanced segmental motion above *T*_g_ drives transient radical contact, outweighing any offsetting increase in average inter-radical distance from thermal expansion (Figure 4d). Activation energies of 1.2–1.9 eV, extracted from these Arrhenius plots, are high, reflecting large reorganization energies characteristic of TEMPO-based radical polymers, arising from charge localization and the conformational demands of achieving effective radical–radical orientation.^45,46^ This behavior contrasts with the temperature-independent electron transfer reported at the single-molecule scale in TEMPO-containing oligomers, where interfacial effects at gold electrodes are proposed to stabilize a high-conductance conformation and electron tunnelling becomes the operative mechanism.^47^

Given the role of chain flexibility in governing radical polymer conductivity, depressing *T*_g_ provides a practical route to enhance performance. P­(GTEMPO-*alt*-GA), the lowest-*T*_g_ sample in the series (<20 °C), retains sufficient mechanical integrity to form a handleable film despite *M*_n_ below the entanglement threshold (Figure S25). We propose this cohesion reflects dipolar interactions between the N–O^•^ groups (∼3 D dipole moment), which act as physical cross-links. Disrupting these interactions should depress *T*_g_ and improve conductivity. Indeed, blending with 10 wt % P­(GTEMPO-*alt*-PA) dropped *T*_g_ from 17 to 4 °C, giving up to 7-fold higher conductivity than neat P­(GTEMPO-*alt*-GA) at 50 °C and extending the measurable conductivity window to 30 °C, where the neat polymer yields no reliable signal (Figure 5a). This is consistent with P­(GTEMPO-*alt*-PA) acting as a plasticizer that disrupts intermolecular radical contacts. At 20 and 30 wt %, *T*_g_ values more closely follow Fox-equation predictions for miscible blends, and conductivity decreases accordingly as the rigid aromatic spacer dilutes the mobile radical network.

**Figure 5:**
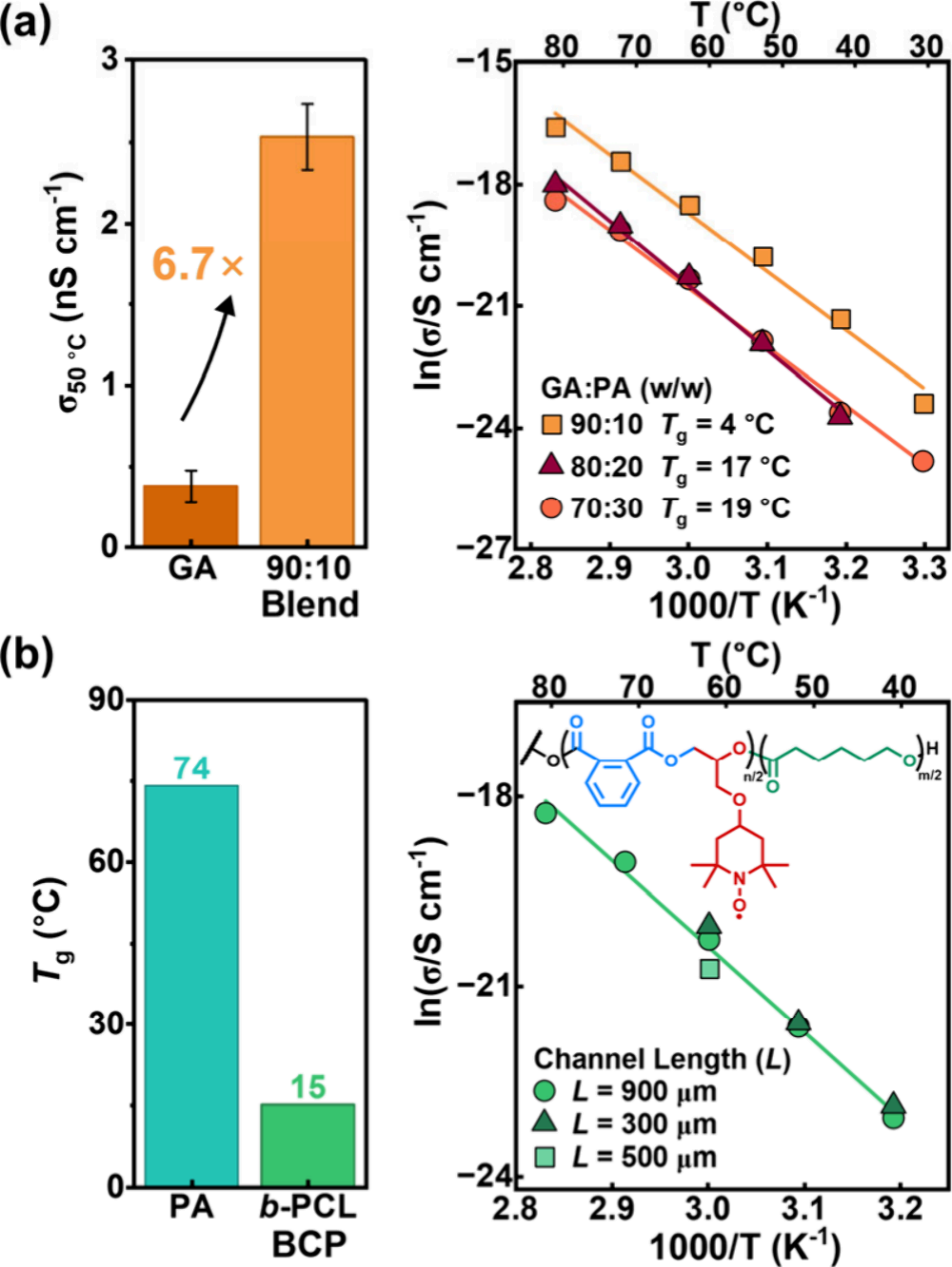
(a) Conductivity enhancement for P­(GTEMPO-*alt*-GA) blended with 10 wt % P­(GTEMPO-*alt*-PA); Arrhenius plots show extended temperature window to 30 °C, with 20 and 30 wt % blends for comparison (*E*_a_ = 1.25 eV). (b) *T*_g_ depression of P­(GTEMPO-*alt*-PA) by block copolymerization to PCL-*b*-P­(GTEMPO-*alt*-PA)-*b*-PCL (34 wt % PCL, inset; *n* = 17, *m* = 30); Arrhenius plots at varying film thicknesses (*E*_a_ = 1.17 eV).

A complementary route to *T*_g_ depression without physical blending is copolymerization. We demonstrated this for P­(GTEMPO-*alt*-PA), incorporating a low-*T*_g_ biodegradable copolyester block, polycaprolactone (PCL). Using the chemoselective one-pot ROCOP/ROP process of Romain et al.,^48^ PCL-*b*-P­(GTEMPO-*alt*-PA)-*b*-PCL triblock copolymer was accessed (Figures 5b and S26–S30). Incorporating 34 wt % PCL depressed *T*_g_ by 80% from the neat radical polymer to 15 °C, with conductivities comparable to P­(GTEMPO-*alt*-GA) of similar *T*_g_. This reinforces chain mobility as the primary determinant of bulk charge transport. The block copolymer formed flexible films with mechanical integrity confirmed by dynamic mechanical analysis (Figure S29). At thicknesses down to 300 μm, conductivity remained invariant, consistent with a bulk transport regime above the critical channel-length thresholds mentioned above. While low *T*_g_ favors conductivity here, glassy films can be advantageous for device fabrication: backbone rigidification of a PTEO analogue (raising *T*_g_ by over 100 °C) recently enabled >95% device viability in memristor arrays.^49^ Block architectures accessible through ROCOP thus offer a route to combine soft conductive and rigid processable segments in a single chain.

In summary, epoxide-anhydride ROCOP provides a route to TEMPO-functional radical polyesters with spacers ranging from flexible aliphatic and heteroatom-containing to rigid alicyclic and semiaromatic. DFT reveals that rigid aromatic spacers bring radical sites into closer through-space proximity than flexible aliphatic analogues of comparable atom counts. Nonetheless, bulk solid-state conductivity across the series establishes *T*_g_ as the primary determinant of charge transport, with spacer geometry mattering principally through its effect on *T*_g_ rather than through direct electronic coupling. The thioether-containing polyester is an exception, where its reduced radical content suppresses the conductivity independently of *T*_g_. This is directly tested by deliberate *T*_g_ depression through blending and block copolymerization: in both cases, conductivity tracks *T*_g_, confirming it as a transferable design handle. Establishing *T*_g_ as a central design lever, independent of the strategy used to set it and prior to any partial oxidative doping enhancements, simplifies the radical polymer design problem, while the sustainable, degradable backbones introduced here broaden the material palette for flexible radical polymer electronics.

## Supplementary Material



## Data Availability

DFT output files are available at https://github.com/GeorgeLGregory/Radical-Polyesters-ROCOP-Spacer-Conductivity.

## Notes

Materials and instrumentation; Polyester synthesis and characterization (^1^H, ^13^C and 2D NMR, SEC, MALDI-ToF MS, TGA); SQUID data; *I*–*V* curves; Blend and block copolymer synthesis and characterization; Computational details (PDF)
