# Supplementary material for: Radical Polyesters: Connecting Spacer Structure to Bulk Electrical Conductivity
Source: ACS Macro Lett. 2026 Jun 18;15(7):1013–20. doi: 10.1021/acsmacrolett.6c00245 (PMC13394413; doi:10.1021/acsmacrolett.6c00245)
Supplement: Supplementary file 1 [file mz6c00245_si_001.pdf]

Radical Polyesters: Connecting Spacer Structure to Bulk Electrical Conductivity

Kieran G. Stakem,[a] *Simon J. Cassidy,*[b] *William K. Myers,*[c] *Georgina L. Gregory* [a,*]

[a] Department of Chemistry, University of Oxford, 12 Mansfield Road, Oxford, OX1 3TA, UK

[b] Department of Chemistry, University of Oxford, South Parks Road, Oxford, OX1 3QR, UK

[c] Centre for Advanced Electron Spin Resonance (CAESR), Department of Chemistry, University of Oxford, South Parks Road, Oxford, OX1 3QR, UK

*Email: georgina.gregory@chem.ox.ac.uk

Table of Contents

Materials and Reagents **3**
Synthetic Procedures **3**
Synthesis of 4-Glycidyloxy-2,2,6,6-tetramethylpiperidine 1-Oxyl (GTEMPO) 3
General procedure for GTEMPO-cyclic anhydride ROCOP 3
Table S1. ROCOP Conditions for polyesters in Scheme 1 4
Scheme S1. General proposed mechanism for epoxide-anhydride ROCOP. 5
Instrument Details **5**
SEC Traces **9**
Figure S2. SEC traces for polyesters in Scheme 1 9
MALDI-ToF Mass Spectrometry **10**
Figure S3. MALDI-ToF mass spectra for rigid-spacers 10
Figure S4. MALDI-ToF mass spectra for flexible-spacers 11
NMR Characterization **12**
Figure S5. ^{1}H NMR spectra before and after paramagnetic TEMPO radical quenching. 12
Figure S6. ^{1}H, ^{13}C{^{1}H} and 2D NMR spectra (DMSO-d_6) for P(GTEMPO-*alt*-MPA). 13
Figure S7. ^{1}H, ^{13}C{^{1}H} and 2D NMR spectra (DMSO-d_6) for P(GTEMPO-*alt*-PA) 14
Figure S8. ^{1}H, ^{13}C{^{1}H} and 2D NMR spectra (DMSO-d_6) for P(GTEMPO-*alt*-HHPA) 15
Figure S9. ^{1}H, ^{13}C{^{1}H}, 2D NMR spectra ($CDCl_3$) for P(GTEMPO-*alt*-TCA). 16
Figure S10. ^{1}H, ^{13}C{^{1}H}, 2D NMR spectra (DMSO-d_6) for P(GTEMPO-*alt*-TDGA). 17
Figure S11. ^{1}H, ^{13}C{^{1}H}, 2D NMR spectra (DMSO-d_6) for P(GTEMPO-*alt*-DGA). 18
Figure S12. ^{1}H, ^{13}C{^{1}H}, 2D NMR spectra (DMSO-d_6) for P(GTEMPO-*alt*-GA). 19
Figure S13. ^{1}H, ^{13}C{^{1}H}, 2D NMR spectra (DMSO-d_6) for P(GTEMPO) (i.e. PTEO) 20
Hydrolytic Degradation Studies **21**
Figure S14. Proof of principle degradation for radical polyesters 21
Density Functional Theory (DFT) **22**
Table S2. Optimised Geometries for N···N spacing and SOMO energy levels. 22
Figure S15. Natural Bond Orbital (NBO) charges on bridging atom for flexible spacers. 23
Thermogravimetric Analysis (TGA) **25**
Figure S17. TGA and derivative (DTG) curves for radical polyesters 25
SQUID Magnetometry – Curie-Weiss Analysis **26**
Figure S18. Zero-field cooled (ZFC) and field-cooled (FC) Curie-Weiss curves 27
Table S3. SQUID magnetometry data for radical content determination (Figure 3b) 27
Figure S19. χ and χ^{-1} as a function of T for P(GTEMPO-*alt*-anhydrides) 28
Figure S20: χT vs. T for rigid and flexible spacers 28
Electron Paramagnetic Resonance (EPR) Spectroscopy **29**
Table S4. Comparison of theoretical and observed spin concentrations by EPR. 29
FT-IR Spectrum **30**
Figure S21. ATR-FTIR spectra for P(GTEMPO-*alt*-GA) 30
Cyclic Voltammetry **30**
Figure S22. Cyclic voltammograms 30
Solid-State Electrical Conductivity **31**
Figure S23. Nyquist plots from EIS and equivalent-circuit fits for rigid and flexible spacers. 31
Figure S24. I-V curves from LSV at temperatures above T_g for rigid spacers 32
Figure S25. I-V curves from LSV at temperatures above T_g for flexible spacers 33
Blend Study **34**
Preparation Procedure 34
Figure S26. DSC traces, solid-state LSV and CV characterisation of blends 34
PCL-*b*-P(GTEMPO-*alt*-PA)-*b*-PCL **35**
Synthetic procedure 35
Figure S27. Synthesis and characterization of block copolymer 35
Figure S28. ^{1}H, ^{13}C{^{1}H} and 2D NMR spectra (DMSO-d_6) of block copolymer 37
Figure S29. DSC, TGA and DMTA of PCL-b-P(GTEMPO-alt-PA)-b-PCL 38
Figure S30. I-V curves for PCL-*b*-P(GTEMPO-*alt*-PA)-*b*-PCL 38
References **39**

Materials and Reagents

All solvents and reagents were obtained from commercial sources (Sigma-Aldrich, TCI, ThermoFisher) and used as received unless stated otherwise. Anhydrous 2-methyltetrahydrofuran (2-MeTHF) and *t*-Bu-P_2 catalyst (~2.0 M in THF) were purchased from Sigma-Aldrich. The following cyclic anhydrides were purchased from TCI: phthalic anhydride (PA, >98.0%), hexahydrophthalic anhydride (HHPA, >99.0%), methylphthalic anhydride (MPA, >98.0%), glutaric anhydride (GA, >98.0%), and diglycolic anhydride (DGA, >98.0%). Thiodiglycolic anhydride (TDGA, 98%) was purchased from ThermoFisher. 4-Glycidyloxy-2,2,6,6-tetramethylpiperidine 1-Oxyl (GTEMPO) was synthesised according to a modified literature procedure (see below) or purchased from TCI (>95.0%). Tricyclic anhydride (TCA) was prepared according to a literature procedure,[1] then recrystallized from hexane and sublimed under vacuum at 125 °C prior to use. 1,4-Benzenedimethanol (BDM; Sigma-Aldrich) was purified by recrystallization from dry toluene (× 3). For block copolymer synthesis, PA was purified by stirring in toluene, cannula filtration, recrystallisation from chloroform, and vacuum sublimation, and ε-caprolactone (Sigma-Aldrich) was dried over CaH_2 and vacuum distilled.

Synthetic Procedures

Synthesis of 4-Glycidyloxy-2,2,6,6-tetramethylpiperidine 1-Oxyl (GTEMPO)

The monomer was synthesised according to a modified literature procedure.[2] A stirring mixture of *(±)*-epichlorohydrin (29.5 mL, 380 mmol, 5.4 equiv.), tetrabutylammonium hydrogensulfate (1 g, 2.9 mmol, 0.04 equiv.), and 50% (w/w) aqueous sodium hydroxide (50 mL) was prepared, and 4-hydroxy-TEMPO (12 g, 70 mmol, 1 equiv.) was slowly added. After stirring overnight, the mixture was poured into ice/water (250 mL), and the aqueous phase extracted with diethyl ether (135 mL). The organic phase was then washed with DI water (75 mL), brine (50 mL), and dried over $MgSO_4$ before removing the solvent *in vacuo* to obtain a red oil. The oil was purified by vacuum distillation: an initial near-colourless fraction was separated off at ~85 °C and 8 x 10^{-1} mbar whilst the subsequent fraction collected at ~140 °C and <8 x 10^{-1} mbar was recrystallized from dry hexane to give the product as a red crystalline powder (6.69 g, 42 %).

General procedure for GTEMPO/cyclic anhydride ROCOP

For GTEMPO/GA: In a glovebox, BDM (65 mg, 0.47 mmol, 1 equiv.) was loaded into an oven-dried vial, followed by 2-MeTHF (2.6 mL), *t*-Bu-P_2 (23 µL, ~2.0 M in hexanes, 0.05 mmol, 0.1 equiv.), GA (0.547 g, 4.7 mmol, 10 equiv.), and GTEMPO (1.06 g, 4.7 mmol, 10 equiv.). The reaction mixture was transferred to a preheated oil bath at 60 °C. Aliquots were periodically withdrawn under an inert atmosphere, quenched with benzoic (or acetic) acid (ca. 1 mg) and monitored by ^{1}H NMR spectroscopy ($CDCl_3$). Upon near-complete anhydride conversion (ca. > 95%), the polymerization was quenched by the addition of benzoic acid (ca. 3 equiv. relative to catalyst). The mixture was then concentrated, redissolved in a minimum volume of DCM,

precipitated into cold isopropyl alcohol (IPA) and centrifuged. Purification was repeated until unreacted monomer and catalyst were no longer detectable by ^{1}H NMR. The polymer was dried under vacuum with heating until no solvent residues were observable by ^{1}H NMR, supported by TGA.

Polymer Purification: Precipitation solvent was selected according to solubility: diethyl ether removed residual GTEMPO monomer (used in slight excess), and alcohols (isopropanol, IPA or methanol) removed both GTEMPO and the *t*-Bu-P_2 catalyst. IPA was preferred over methanol because the polymers were less soluble in it, minimising product loss. For PA, GA, HHPA, and MPA, the procedure was as described above. DGA and TDGA were precipitated sequentially into diethyl ether, diethyl ether/IPA, and neat IPA; precipitations were performed cold (freezer or cryogen) to minimise product loss. For TCA, where alcohol solubility precluded precipitation, purification was performed using a silica plug.

Table S1. ROCOP Conditions for polyesters in Scheme 1.

Anhyd.	BDM (mg)	Anhyd. (g)	GTEMPO (g)	P2 (µL)[a]	Equiv. (BDM = 1)			T (°C)[c]	2-MeTHF (mL)[d]
					GTEMPO	Anhyd.	P2		
**PA**	43	0.45	0.70	156	10	10	1.0	60	1.7
**MPA**	26	0.30	0.50	94	12	10	1.0	60	1.1
**TCA**	63	1.20	1.40	23	14	12	0.1	70	2.4
**HHPA**	63	0.80	1.30	23	13	11	0.1	60	2.4
**GA**	65	0.55	1.10	23	10	10	0.1	60	2.6
**DGA**	40	0.34	0.72	14	11	10	0.1	75	1.6
**TDGA**	25	0.25	0.52	10	13	10	0.1	60	1.0

[a] ca 2.0 M in THF. [b] Epoxide used in slight excess with respect to anhydride (1.1 ± 0.1 equiv. relative to anhydride) as is common practice for anhydride/epoxide ROCOP given 1st order rate dependence on epoxide and zeroth order on anhydride concentration. [c] Higher temperatures used to increase rate and aid monomer solubility; Reaction times typically, 22.5 h for PA to 64 h for HHPA and > 96 h for TCA and DGA, depending on catalyst loading. [d] 2-MeTHF chosen for its green credentials, but THF and toluene solvents were also tested and achieve similar results; Initial total monomer concentration, $[\mathrm{M}]_0 = \frac{mmol_{GTEMPO} + mmol_{Anhydride}}{mL_{2-MeTHF} + mL_{GTEMPO}}$ = ca. 3 M. GTEMPO has low melting point of 38 °C, ρ taken as 0.91 g mL^{-1}, corresponding to < 3 mL solvent for each polymerization.

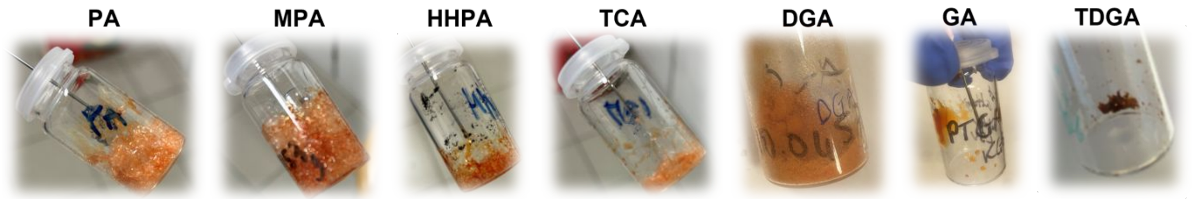

Figure S1. Photographs of representative polyester samples prepared from each anhydride, shown as isolated after drying. The high-T_g polyesters (PA, MPA, HHPA, TCA) were subsequently ground to fine powders for characterisation and are difficult to distinguish visually.

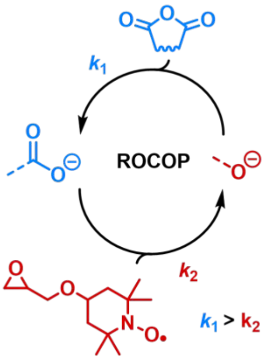

Scheme S1. General proposed mechanism for epoxide-anhydride ROCOP.

Instrument Details

NMR Spectroscopy. ^1H, 2D COSY and HSQC and $^{13}C\{^1H\}$ NMR spectra were obtained at room temperature using either a Bruker Avance III HD nanobay NMR equipped with a 9.4T magnet (400 MHz) or a Bruker Avance III NMR equipped with a 11.75T magnet (500 MHz). ^1H-^1H DOSY NMR spectra were obtained using a Bruker Avance III NMR equipped with a 11.75T magnet (500 MHz). Samples were prepared in either deuterated chloroform ($CDCl_3$) or deuterated dimethyl sulfoxide (DMSO-d_6).

Size Exclusion Chromatography (SEC). Measurements were performed on two instruments. A Shimadzu LC-20AD instrument using two PSS SDV linear M columns in series was used with THF as eluent at 30 °C and a flow rate of 1 mL min^{-1}. Samples were detected using a photodiode array (PDA) detector at 254 nm and/or a refractive index (RI) detector. An Agilent LC1260 Infinity II System fitted with a PLgel 5 µm (50 x 7.5 mm) guard column and two PLgel 5 µm MIXED-C (300 x 7.5 mm) columns connected in series was used with THF as eluent at 35 °C and a flow rate of 1 mL min^{-1}, with RI detection. For both methods, number-average molar mass ($M_{n,SEC}$) and dispersity ($Đ = M_w/M_n$) were determined against narrow polystyrene calibrants. Samples were filtered through 0.2 µm membrane filter prior to injection.

Differential Scanning Calorimetry (DSC). Measurements on the seven anhydride homopolymers were performed on a DSC25 (TA Instruments) using aluminium pans with a pinhole in the lid. Measurements on the blend and block copolymer samples were performed on a DSC2500 (TA Instruments) using hermetically sealed aluminium pans. Both instruments were calibrated with indium, with a sealed empty pan as reference. Samples were cooled under N_2 flow from room temperature to −80 °C, then heated to 150 °C (first heating cycle), cooled to −80 °C, and heated to 150 °C twice more (second and third heating cycles). All heating and cooling rates were 10 °C min^{-1}. Glass transition temperatures (T_g) were taken as the midpoint of the transition in the third heating cycle.

Thermogravimetric Analysis (TGA). Measured were performed using a TGA5500 system (TA Instruments). Polymer samples were placed on platinum plates and heated from ambient temperature to 450 °C at 10 °C min^{-1} under N_2 flow.

MALDI-ToF Mass Spectrometry. Spectra were recorded on a Bruker Autoflex Speed MALDI-TOF instrument in positive ion linear mode. Samples were dissolved in THF (10 mg mL^{-1}). DCTB matrix (10 mg mL^{-1} in THF) and KTFA salt (10 mg mL^{-1} in THF) were used. The three components were mixed in approximately equal volumes, spotted onto a ground stainless steel Bruker MALDI plate, and allowed to air dry before analysis. High laser power (*ca.* 90%) was typically required.

LC-MS: High-resolution mass spectra of polymer degradation products were recorded on a Waters LCT Premier XE orthogonal acceleration time-of-flight mass spectrometer operated in direct infusion (loop injection) mode without front-end chromatographic separation. Samples were introduced via a Waters 1525µ binary HPLC pump coupled to a Waters/CTC Analytics 2777C sample manager. Spectra were injected in water and acquired in positive electrospray ionisation mode over a mass range of 0 to 6000 m/z.

Fourier-Transform Infrared Spectroscopy (FTIR). Spectra were recorded on a Shimadzu IRSpirit spectrometer fitted with a KBr window and temperature-controlled DLATGS detector. Measurements were performed inside a glovebox using a single-reflection ATR accessory and acquired over 4700 to 340 cm^{-1} at a 4 cm^{-1} resolution with 100 scans co-added per spectrum.

Superconducting Quantum Interference Device (SQUID). Measurements were performed using an MPMS-3 magnetometer. Samples of 5 – 15 mg were prepared in gelatin capsules, with sections of plastic straw used to secure them in place. Field-cooled measurements were made on warming from 2 to 300 K with an applied field of 1000 Oe. A heating rate of ~ 6 min K^{-1} was used over $2 \leq T < 20$ K and ~ 0.33 min K^{-1} over $20 < T < 300$ K.

Electron Paramagnetic Resonance (EPR) Spectroscopy. EPR was collected in the Centre for Advanced ESR (CAESR) in the Department of Chemistry of the University of Oxford. The spectrometer was a Bruker EMXmicro with a Premium bridge and ER4122SHQE-W1 resonator with the sample held at room temperature in an Oxford Instruments ESR900 cryostat. Measurements were performed on polymer solutions in toluene. Typical acquisition conditions were microwave frequency of 9.87 GHz, a 100 kHz magnetic field modulation of 0.1 mT and microwave power of 2.0 mW. Data was acquired under non-saturating conditions and was normalized by resonator Q-factor, field modulation amplitude, and microwave power, in addition to the spectrometer software normalization process. Spin counts were determined on the spectrometer software by double integration of the recorded spectra. Spectra were exported as .csv files using EasySpin open-source MATLAB and plotted in OriginPro.

Linear Sweep Voltammetry (LSV). Prior to measurement, samples were dried under vacuum with heating until no residual solvent was observable by ^{1}H NMR (in $CDCl_3$ due to residual water in DMSO-d_6). Rigid polymers were ground to fine powders to aid solvent removal. The absence of retained water and other volatiles was further supported by TGA, which showed no mass loss below 150 °C. To verify minimal contribution of absorbed atmospheric moisture to the measured conductivities, measurements were performed on all samples stored and handled in an argon-filled glovebox (<0.1 ppm H_2O); these showed no appreciable difference from samples loaded under ambient conditions. Through-plane electrical measurements (current passes perpendicularly through the thickness of the sample) were carried out using a BioLogic enhanced Controlled Environment Sample Holder (CESH-e), a leak-tight enclosure for air- and moisture-sensitive materials that acts as a Faraday cage. Temperature was monitored via a PT1000 probe in the CESH-e chamber and controlled via either a BioLogic ITS-e unit or a Binder oven fitted with a side port and thermocouple.

Samples were loaded into a through-plane sample holder with symmetric gold electrodes (6.35 mm diameter) that fits into the CESH-e chamber. An insulating spacer (ca. 0.9–1 mm thick, 8 mm diameter to ensure total sample coverage of the electrode area) maintained sample thickness, measured with a micrometer and the BioLogic thickness measurement kit. The sample holder is fitted with a compression spring and pressure screw to apply a reproducible linear compression force of 100 N, aiding good sample-electrode contact regardless of sample material properties. The cell was operated in a four-terminal (Kelvin) configuration: each electrode was connected to both a current-carrying lead (working/counter) and a separate voltage-sensing lead (reference), so that the current-carrying and voltage-sensing connections are independent. As no current flows through the voltage-sensing leads, this excludes the resistance of the leads from the determined sample resistance.

Samples were heated above their T_g to anneal and ensure good contact with the electrodes. Measurements were recorded on a BioLogic SP-150e potentiostat. I-V curves were recorded at 1 mV s^{-1} over +3 to +5 V versus the open circuit voltage (OCV), following at least one hour of stabilisation at OCV at each temperature. Resistance (R) was taken as the reciprocal gradient of the I-V curve following Ohm's Law, and DC electronic conductivity (σ) determined as:

$$\sigma = \frac{L}{A \times R} \quad \textbf{Equation S1}$$

where A is the electrode cross-sectional area (πr^2 = 0.317 cm^2) and L is the sample thickness.

Activation energies (E_a) were extracted by fitting conductivity data (from LSV) to the Arrhenius equation:

$$\sigma = \sigma_0 \exp\left(\frac{E_a}{k_B T}\right) \; ; \; \ln(\sigma) = \ln(\sigma_0) - \frac{E_a}{k_B T} \quad \textbf{Equation S2}$$

where σ is conductivity (S cm^{-1}), σ_0 is the pre-exponential factor, E_a is the activation energy (eV), k_B is the Boltzmann constant (8.617 × 10^{-5} eV K^{-1}), and T is temperature (K). E_a is extracted from the slope ($-E_a/k_B$) of a linear fit to ln(σ) vs 1/T.

Electrochemical impedance spectroscopy (EIS). EIS was used to verify the measured resistance was dominated by the bulk sample response rather than electrode/contact contributions. Spectra were recorded on a BioLogic MTZ-35 impedance analyser with the sample in the same through-plane cell, from 1 MHz to 10 mHz at 10 points per decade with a 10 mV AC perturbation. A single semicircle was observed and fitted to an equivalent circuit in EC-lab (v11.62), giving a bulk resistance consistent with that obtained by DC measurement at the same temperature and confirming negligible contact resistance (Figure S23). EIS was performed at an elevated temperature (dependent on spacer type) so that the sample resistance was low enough for a semicircle to be resolved within the analyser's frequency and impedance range.

Cyclic Voltammetry (CV). Measurements were performed using a PalmSens EmStat Blue potentiostat in a N_2 glovebox, using a three-electrode configuration: a glassy carbon working electrode (2.0 mm^2), a Pt disc counter electrode (2.0 mm^2), and an Ag wire pseudo-reference electrode. Experiments were conducted in anhydrous, degassed acetonitrile (MeCN) containing ca. 0.1 M [t-Bu_4N][PF_6] as the electrolyte. Ferrocene (Fc, ca. 1 mg) was present in solution alongside the polymer analyte (ca. 5 mM polymer TEMPO units), and all potentials are reported relative to the Fc/Fc$^+$ couple (set to 0 V), determined as $E_1/_2 = (E_{pa} + E_{pc})/2$ of the internal ferrocene. As the Ag wire pseudo-reference is subject to drift, each polymer voltammogram was referenced to the ferrocene co-dissolved with it, rather than to Fc measured in the absence of polymer.

Dynamic Mechanical Thermal Analysis (DMTA), Measurements were carried out for the radical all polyester triblock copolymer using a DMA850 (TA Instruments), fitted with an ACS III cooling system. A rectangular specimen was cut from the hot-pressed film and experiments conducted in tension mode from -90 °C to 30 °C at a heating rate of 3 °C min^{-1} and 1 Hz frequency. T_g are reported as the onset of the drop in storage modulus, E'.

SEC Traces

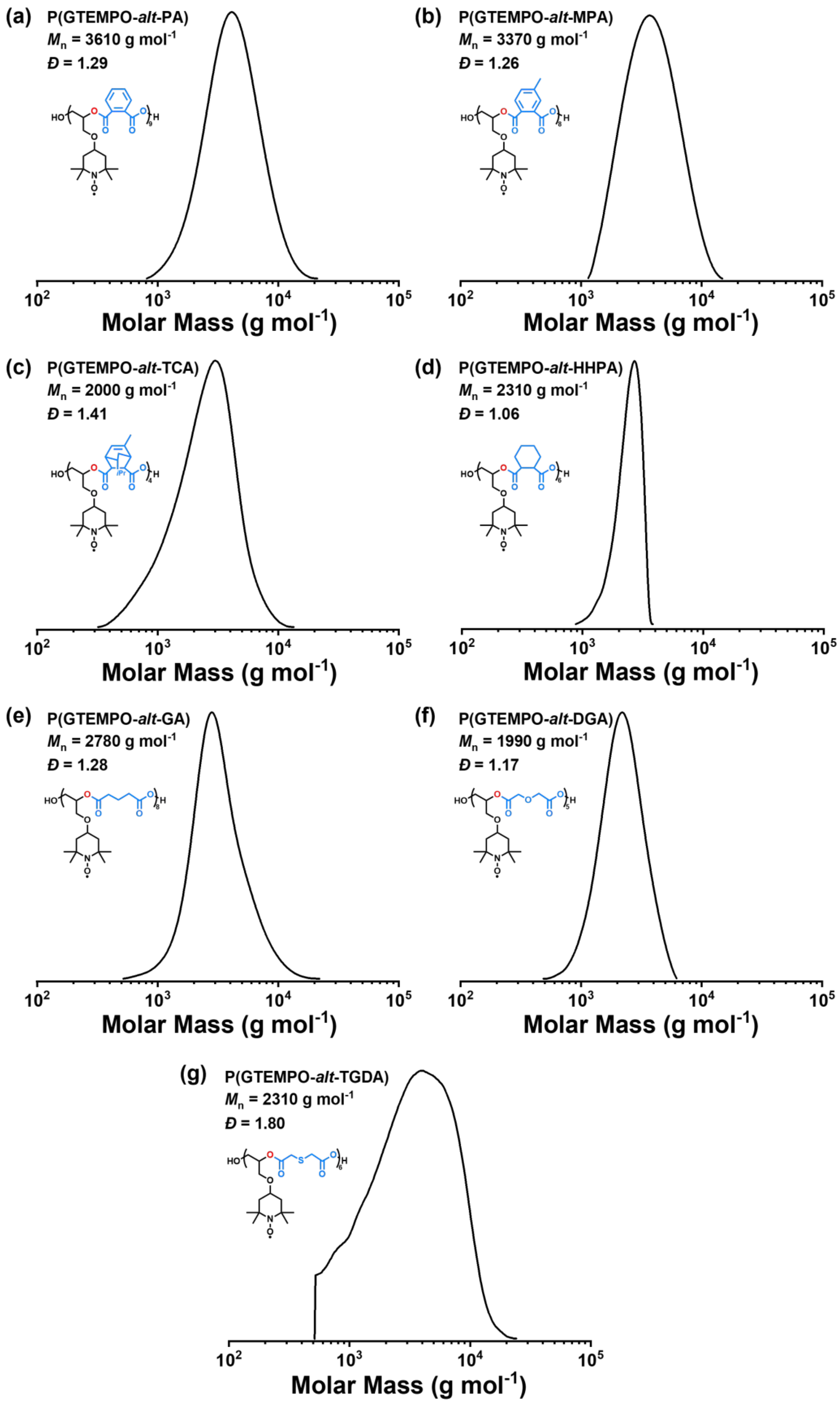

Figure S2. (a)-(g) SEC traces (THF eluent, relative to narrow PS standards) for polyesters in Scheme 1 (UV detector for PA and MPA-based polymers, RI detector for rest). (g) Trace cut due to overlap with solvent peak.

MALDI-ToF Mass Spectrometry

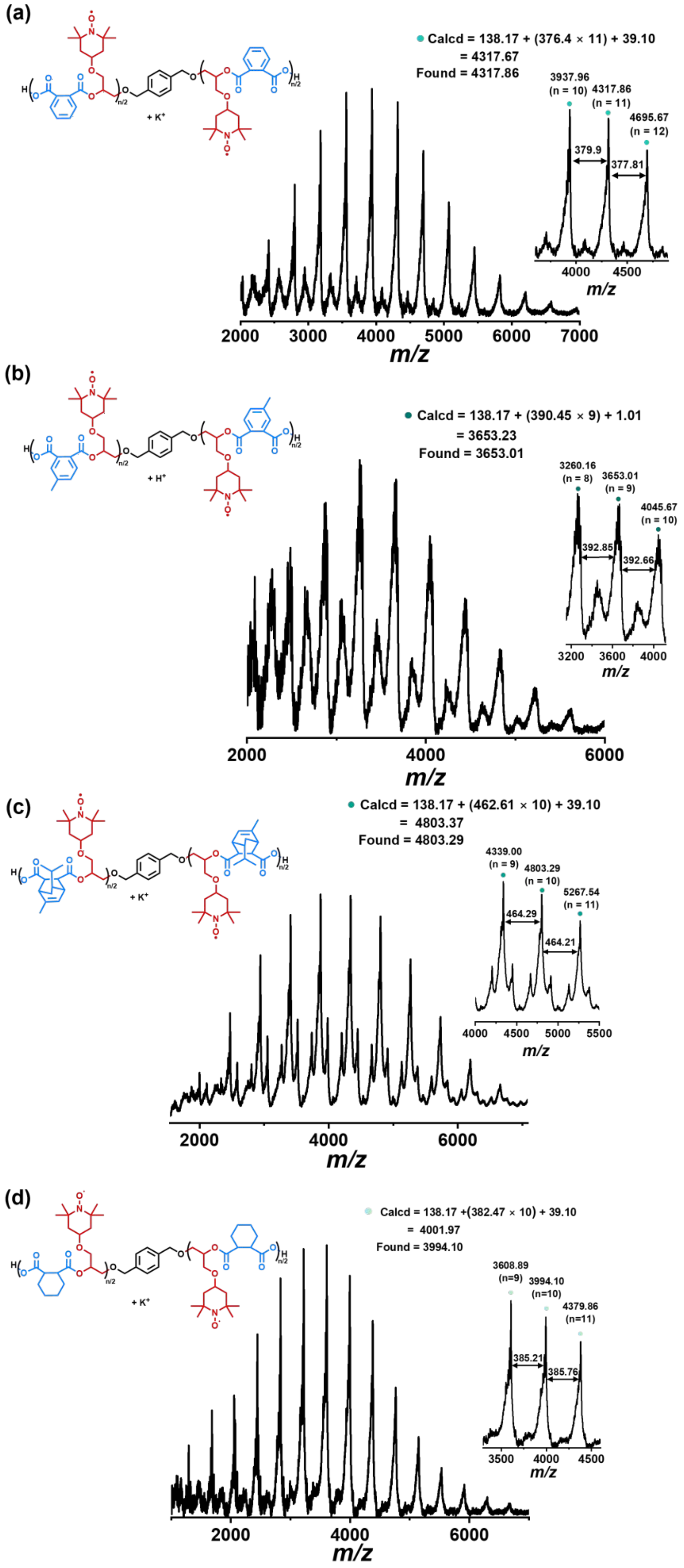

Figure S3. MALDI-ToF mass spectra for rigid-spacer P(GTEMPO-*alt*-anhydrides): (a) PA, (b) MPA, (c) TCA, (d) HHPA. Each panel shows the full spectrum with an expanded region (inset,

right) and the BDM-centred difunctional chain architecture illustrated (inset, left). The observed mass series is consistent with the expected repeat unit mass and BDM end group. An example mass calculation for a representative peak is provided for each polymer. Small systematic offsets of 1–3 Da between observed and calculated repeat unit masses are attributed to instrument calibration and the redox state of the TEMPO radical (TEMPO•/TEMPO-OH) during sample preparation.

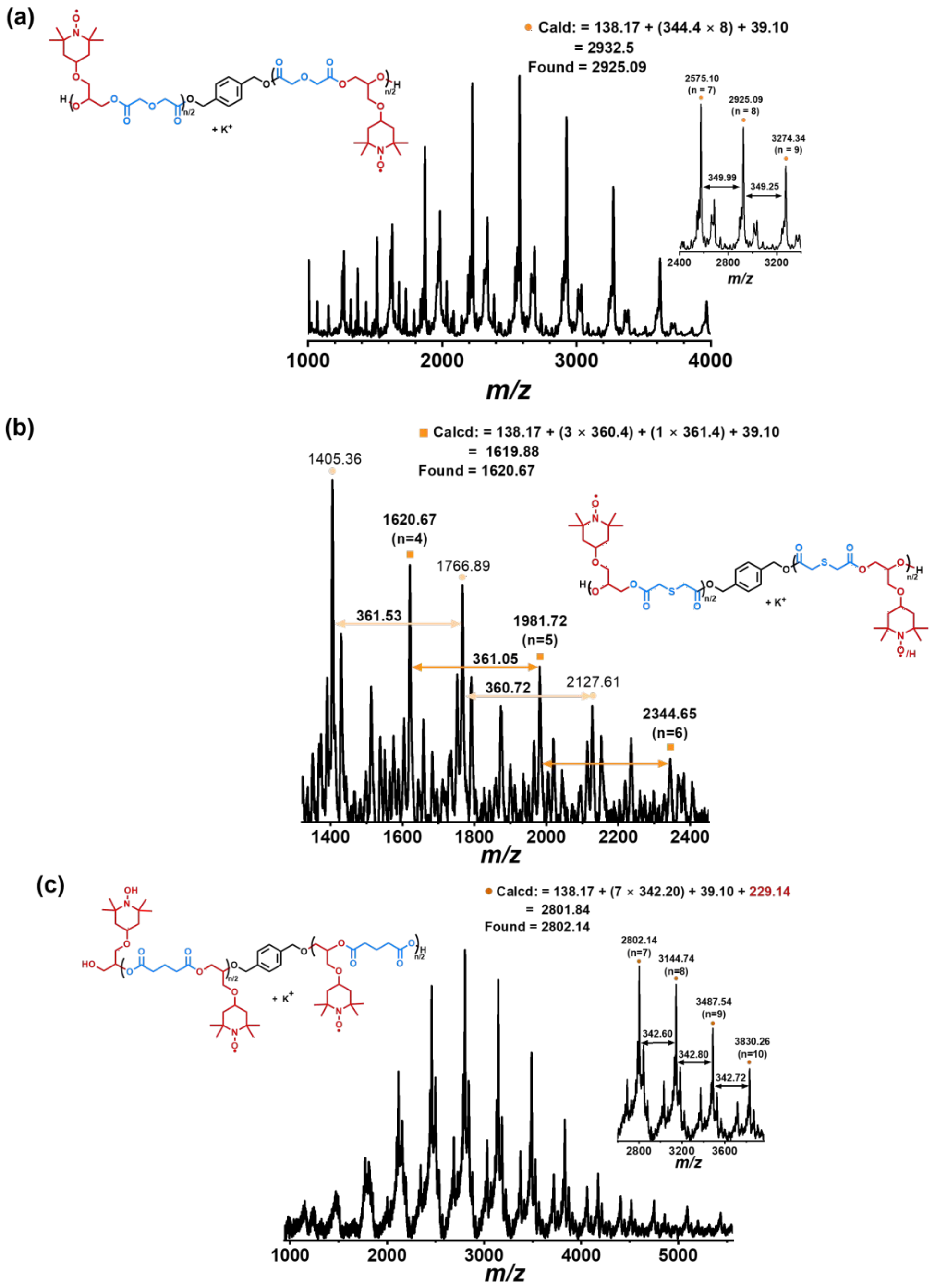

Figure S4. MALDI-ToF mass spectra for flexible-spacer P(GTEMPO-*alt*-anhydrides): (a) DGA, (b) TDGA, (c) GA. Panels (a) and (c) show the full spectrum with an inset expanded region. For P(GTEMPO-*alt*-GA), an additional terminal GTEMPO unit (229.14 *m/z*) is observed. For P(GTEMPO-*alt*-TDGA), data quality was limited; the observed distribution is consistent with DP = 4 (three radical, one hydroxylamine repeat unit), alongside a second unassigned distribution of consistent repeat unit spacing.

NMR Characterization

Pentafluorophenylhydrazine was added (approximately 1:1 by weight) to NMR samples to reduce the nitroxide radicals to the corresponding hydroxylamine, improving resolution of resonances in proximity to the radical centre (Figure S5). Peaks arising from the quenching agent are indicated in the spectra (Q). In general, although all polymers were soluble in $CDCl_3$, clearer peak resolution for assignment was achieved in DMSO-d_6 for all except the TCA-derived polyester. The ether backbone methine environment (proton *f*, see chemical structures below) is sensitive to the anhydride-derived spacer. In DMSO-d_6, this resonance appears at 5.37–5.27 ppm for semi-aromatic polyesters (PA, MPA), at 5.1–5.2 ppm for flexible aliphatic spacers (GA, 5.07 ppm; TDGA, 5.14 ppm; DGA, 5.15 ppm), and at ca. 5.00 ppm for bicyclic, alicyclic systems (TCA, HHPA). As proton *f* is adjacent to the ester oxygen, its chemical shift is expected to reflect the electron density modulated by the anhydride-derived carbonyl. In the semi-aromatic series, conjugation of the carbonyl with the aromatic ring enhances its electron-withdrawing character, depleting electron density on the ester oxygen and deshielding the methine. Flexible aliphatic carbonyls exert moderate electron withdrawal, giving intermediate shifts. For the aliphatic cyclic systems, the rigid ring constrains the carbonyl geometry, reducing conjugation efficiency and affording comparatively greater shielding of the methine.

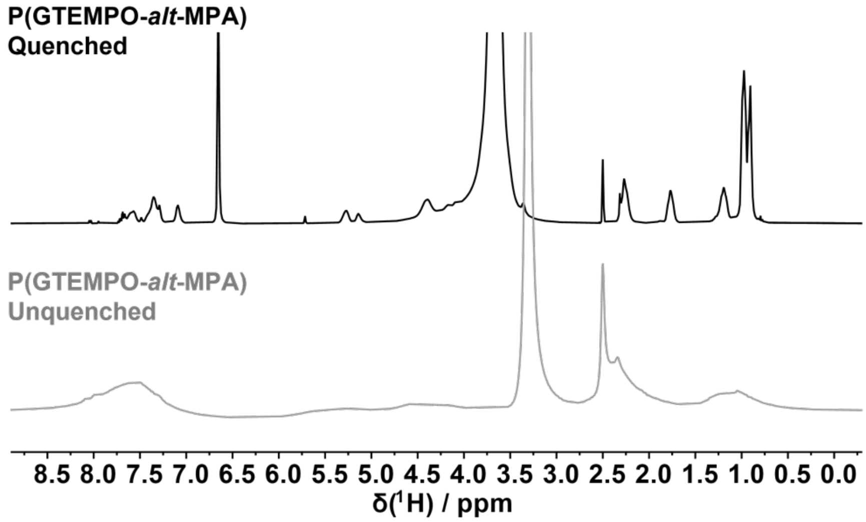

Figure S5. ^{1}H NMR spectra (400 MHz, DMSO-d_6) before and after paramagnetic TEMPO radical quenching. Representative example.

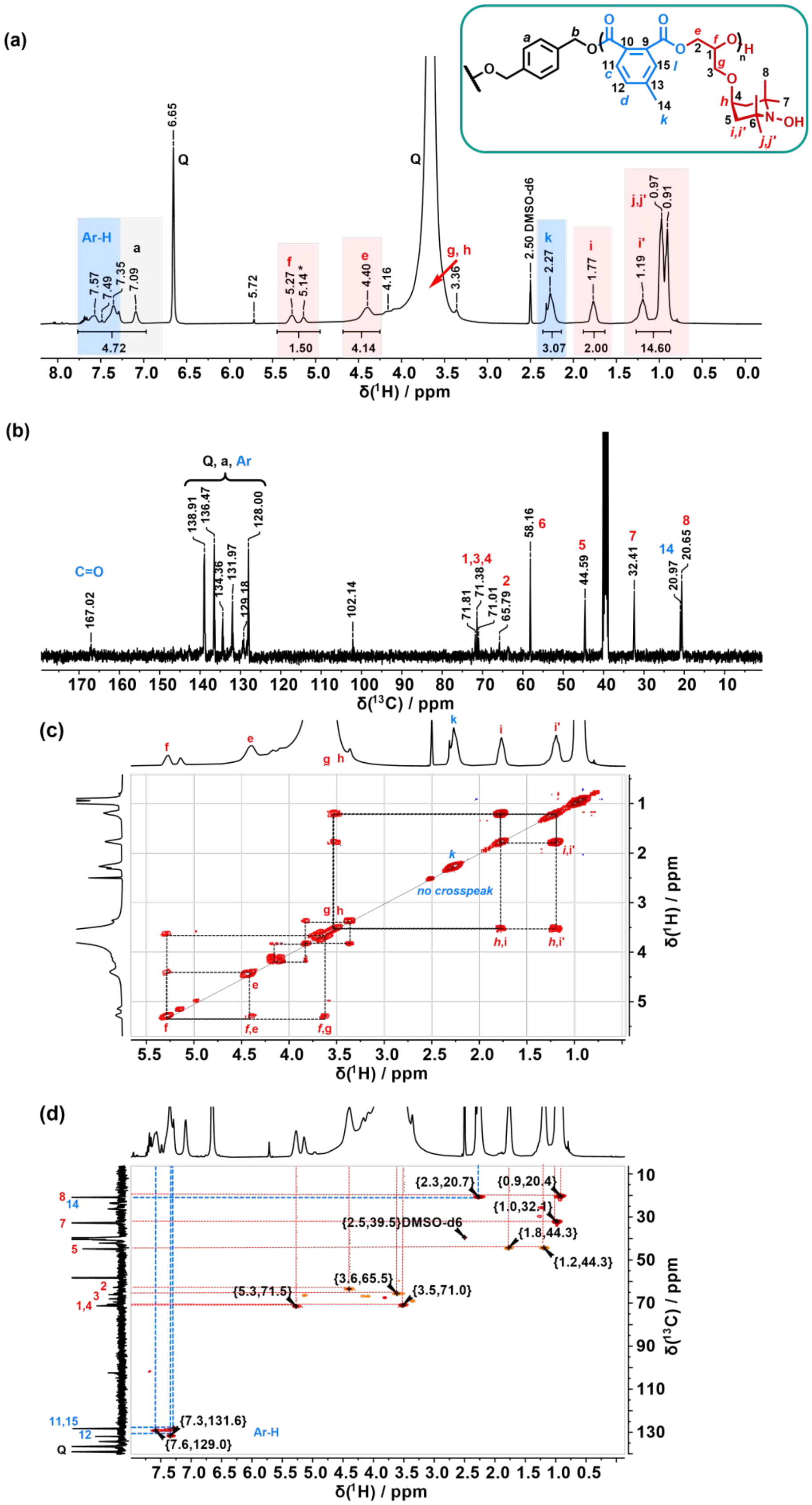

Figure S6. ^{1}H, ^{13}C{^{1}H} and 2D NMR spectra (DMSO-d$_6$) for P(GTEMPO-*alt*-MPA). (a) ^{1}H NMR; *Assigned to TEMPO-OH; Q = peaks due to quenching agent. (b) ^{13}C{^{1}H} NMR; (c) COSY; (d) HSQC; *i* and *i'* protons (1.2 and 1.8 ppm) correlate to the same CH$_2$ (C5 at 44 ppm). Aromatics, Ar-H (C11, C12, and C15) assigned through cross peaks (*c*, *l*, and *d*) in HSQC.

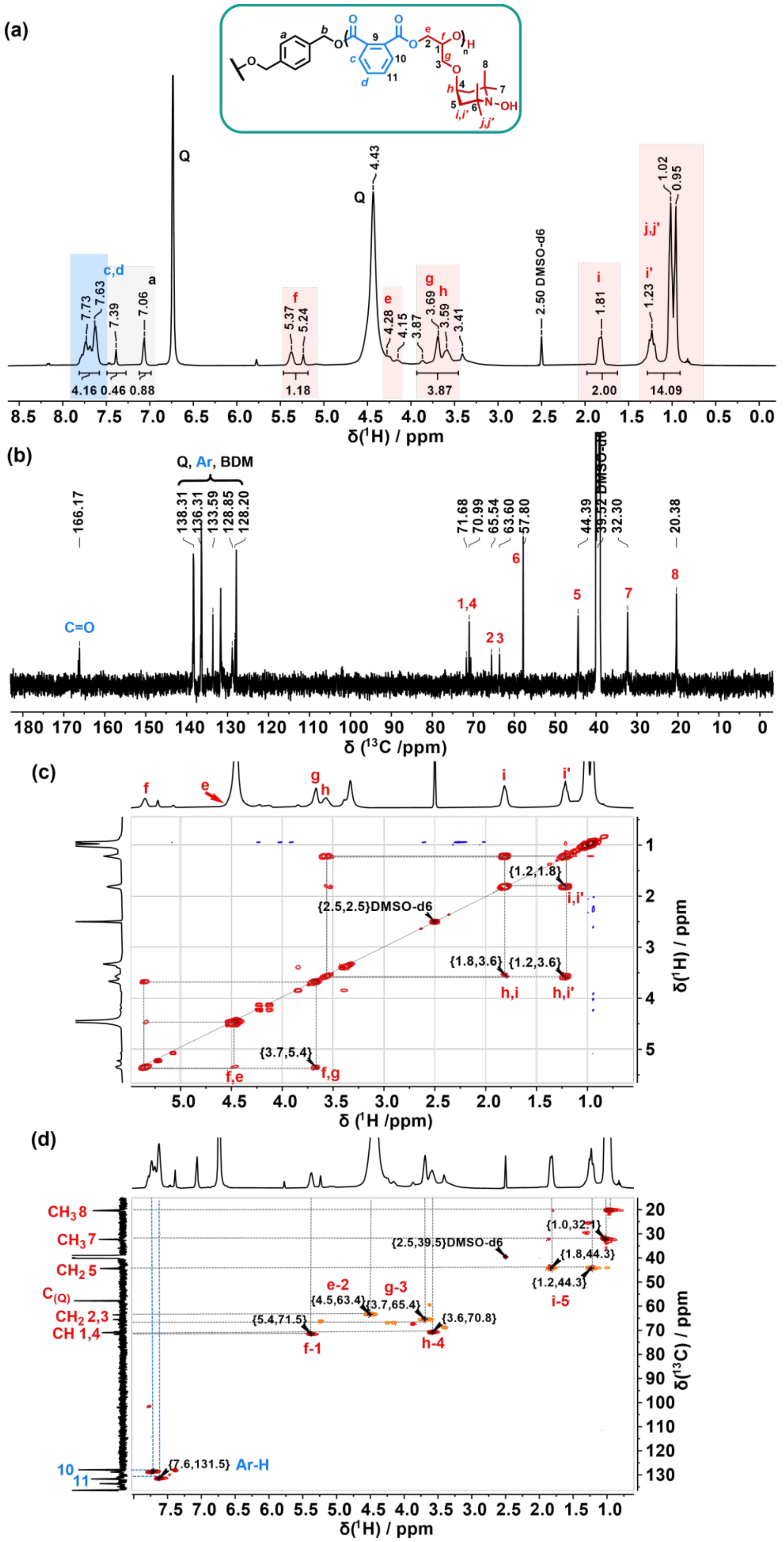

Figure S7. ^{1}H, ^{13}C{^{1}H} and 2D NMR spectra (DMSO-d$_6$) for P(GTEMPO-*alt*-PA). (a) ^{1}H NMR; (b) ^{13}C{^{1}H} NMR; Q = quenching agent, 5.24 ppm attributed to TEMPO-OH. (c) COSY. (d) HSQC; ^{13}C assignments aided by DEPT-135 and DEPT-90 experiments as indicated (*e.g., f, h* at 5.4, 3.6 ppm belong to *CH* and *e, g* at 4.5, 3.7 ppm to CH_2); C(Q) = quaternary carbon.

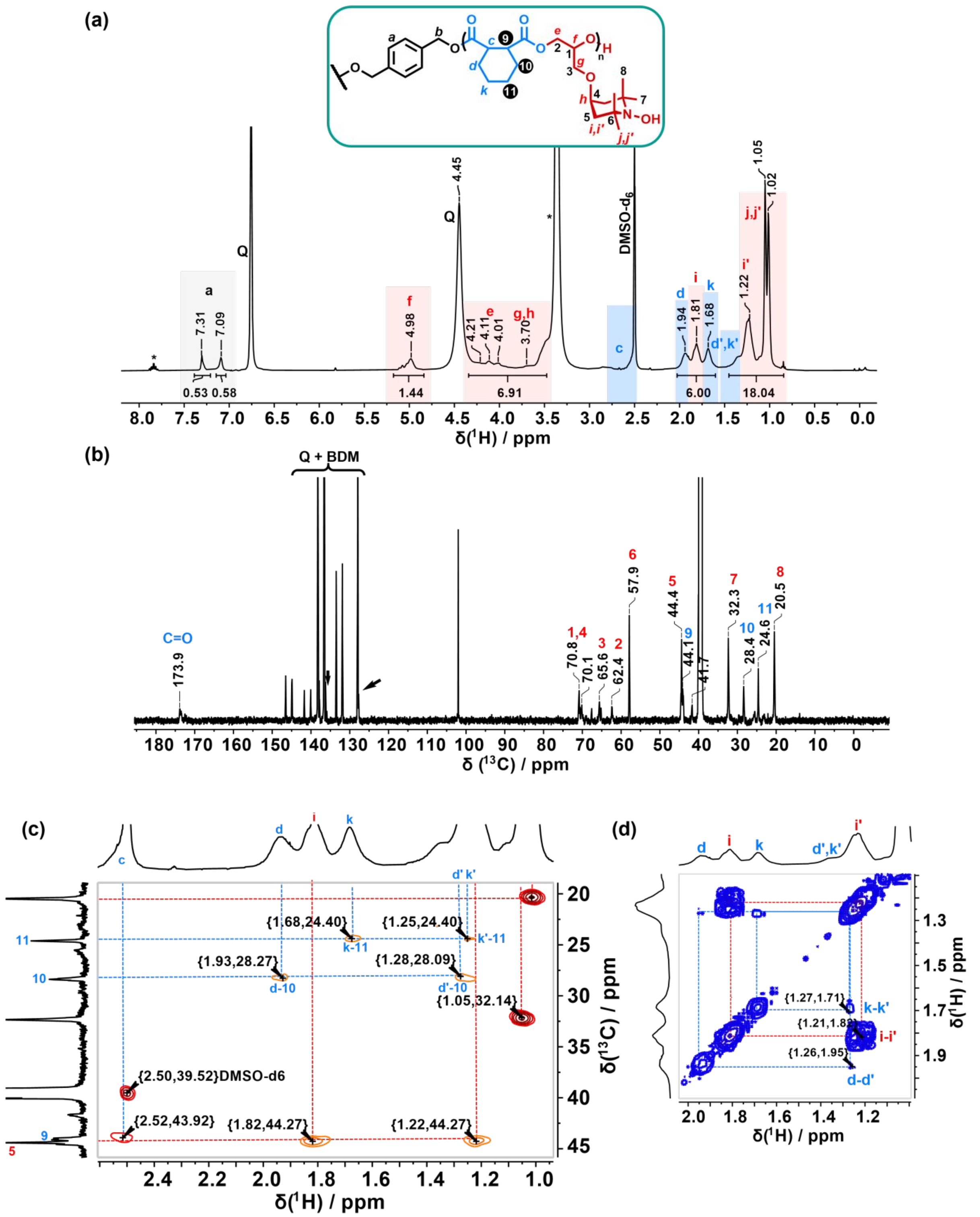

Figure S8. ^{1}H, $^{13}C\{^{1}H\}$ and 2D NMR spectra (DMSO-d$_6$) for P(GTEMPO-*alt*-HHPA). (a) ^{1}H NMR; Q = quenching agent; *impurities from quenching agent and residual water in DMSO-d$_6$ (3.33 ppm). (b) $^{13}C\{^{1}H\}$ NMR; (c) HSQC showing assignment of HHPA ring *d, d'* and *k, k'* protons to the same CH_2 C10 and C11 ^{13}C environments, respectively and *c* to CH environment at ca. 44 ppm. (d) COSY.

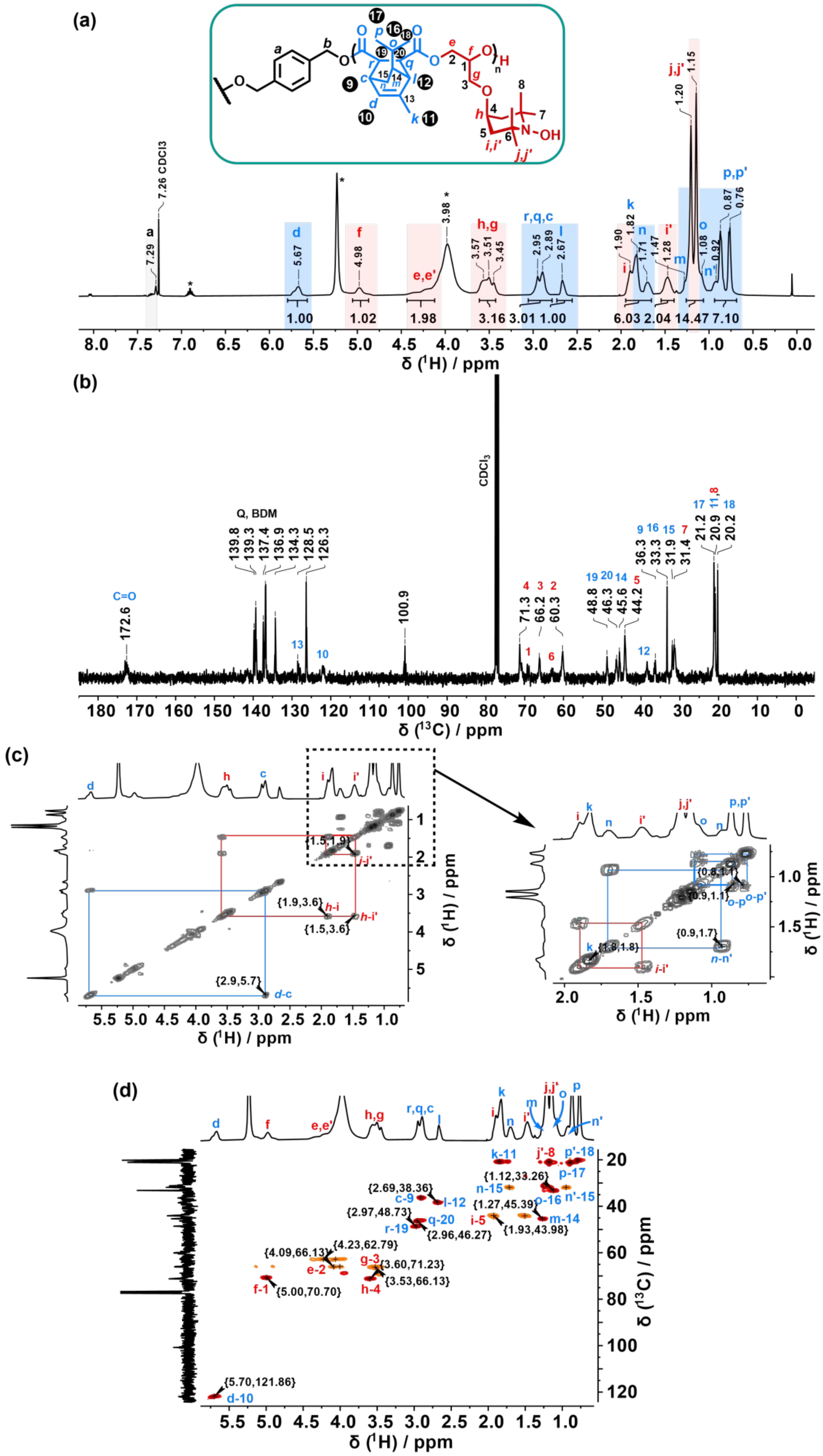

Figure S9. ^{1}H, $^{13}C\{^{1}H\}$, 2D NMR spectra (**$CDCl_3$**) for P(GTEMPO-*alt*-TCA). (a) ^{1}H NMR; *quenching species. (b) $^{13}C\{^{1}H\}$ NMR; (c) COSY with inset region. (d) HSQC aided by DEPT135 and DEPT90 experiments.

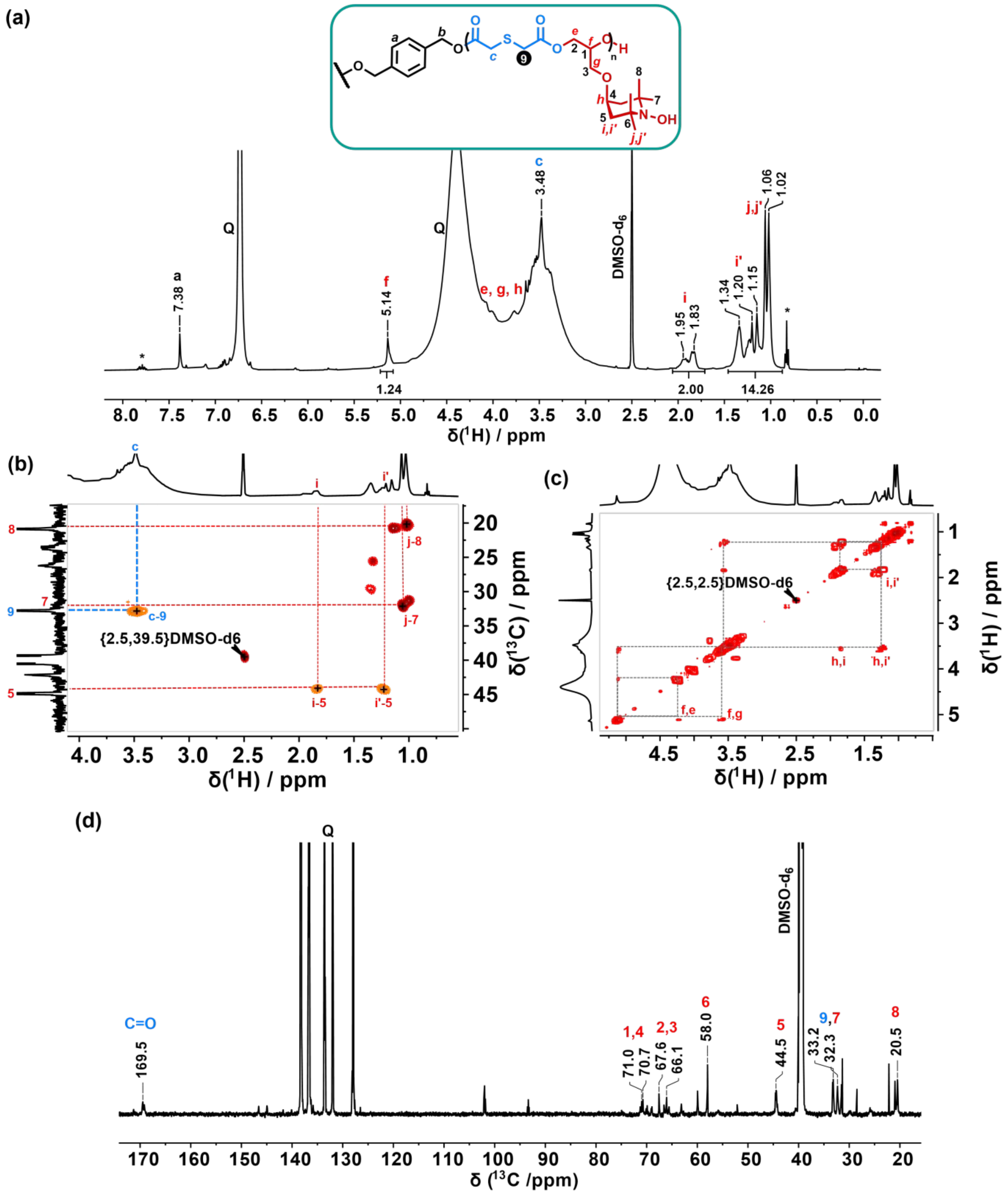

Figure S10. ^{1}H, $^{13}C\{^{1}H\}$, 2D NMR spectra (DMSO-d_6) for P(GTEMPO-*alt*-TDGA). (a) ^{1}H NMR; Q = quenching agent; *impurities in Q and solvent. (b) HSQC showing assignment of *c* protons through correlation to C9. (c) COSY. (d) $^{13}C\{^{1}H\}$ NMR.

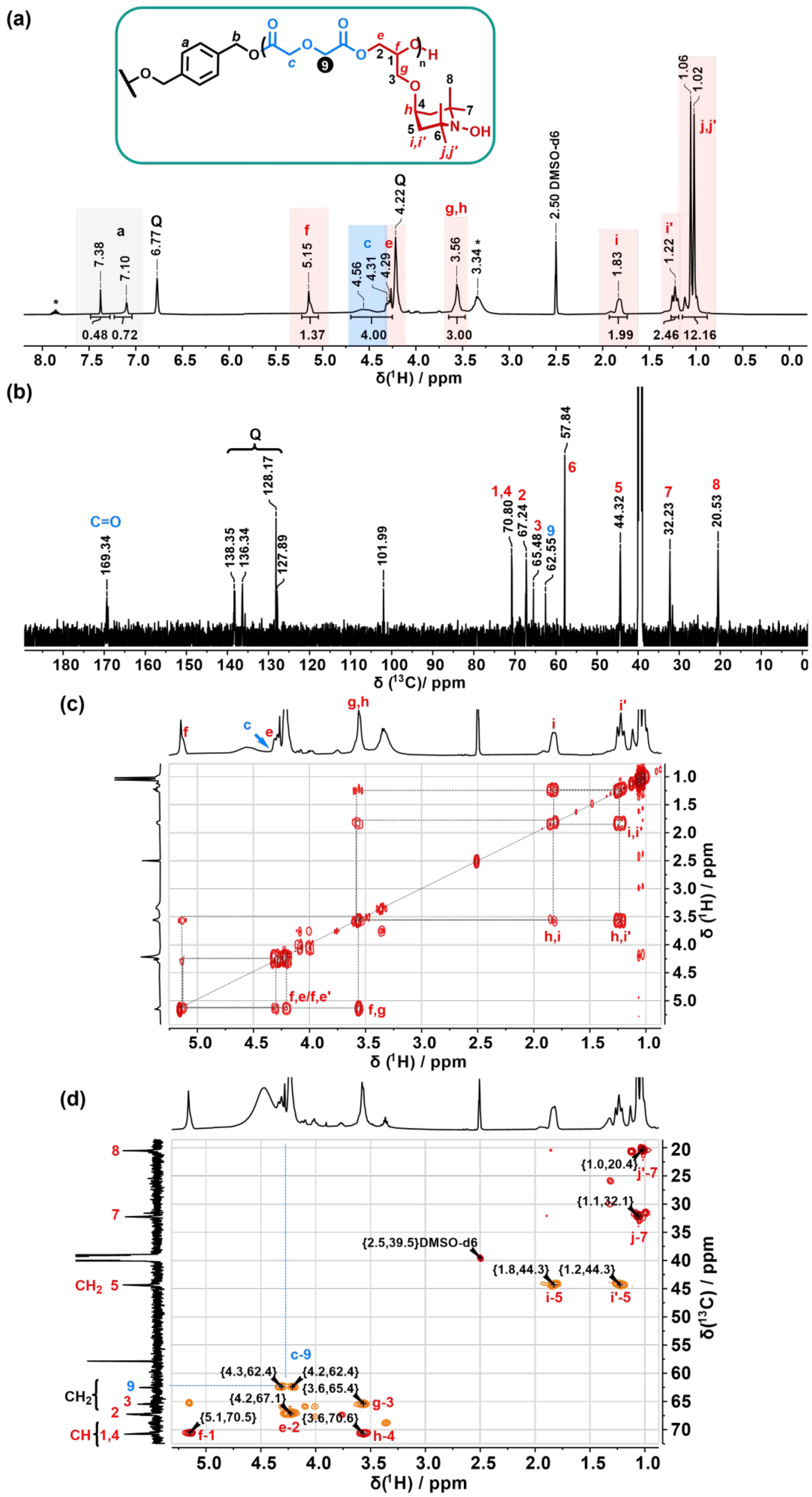

Figure S11. ^{1}H, $^{13}C\{^{1}H\}$, 2D NMR spectra (DMSO-d_6) for P(GTEMPO-*alt*-DGA). (a) ^{1}H NMR; Q = quenching agent; *impurity from Q. (b) $^{13}C\{^{1}H\}$ NMR. (c) COSY. (d). HSQC showing spacer methylene *c* proton assignment through correlation to C9.

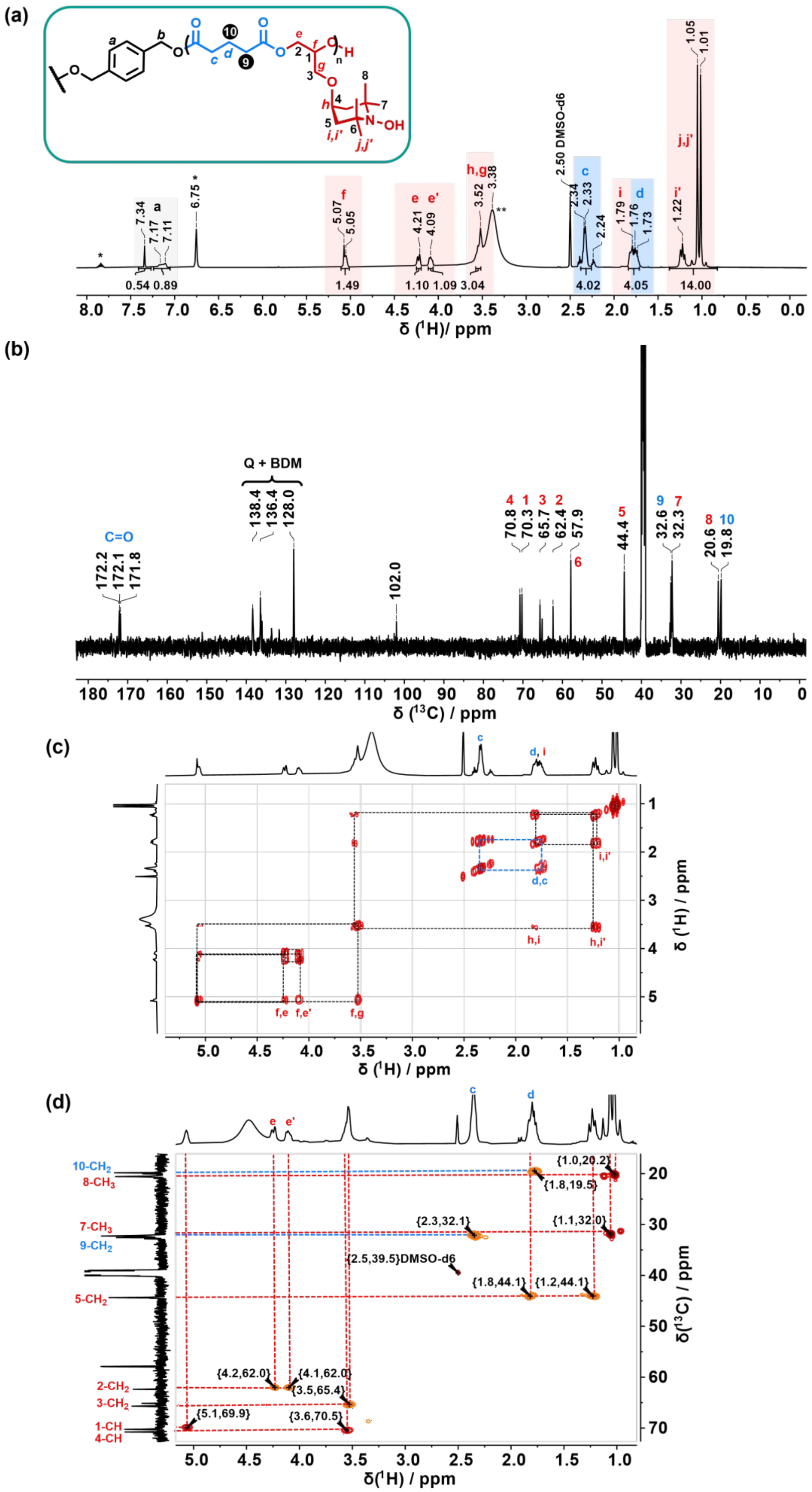

Figure S12. ^{1}H, $^{13}C\{^{1}H\}$, 2D NMR spectra (DMSO-d_6) for P(GTEMPO-*alt*-GA). (a) ^{1}H NMR; *Quenching; **H_2O. (b) $^{13}C\{^{1}H\}$ NMR. (c) COSY. (d) HSQC showing methylene assignments for spacer, aided by DEPT-90 and DEPT-135 experiments.

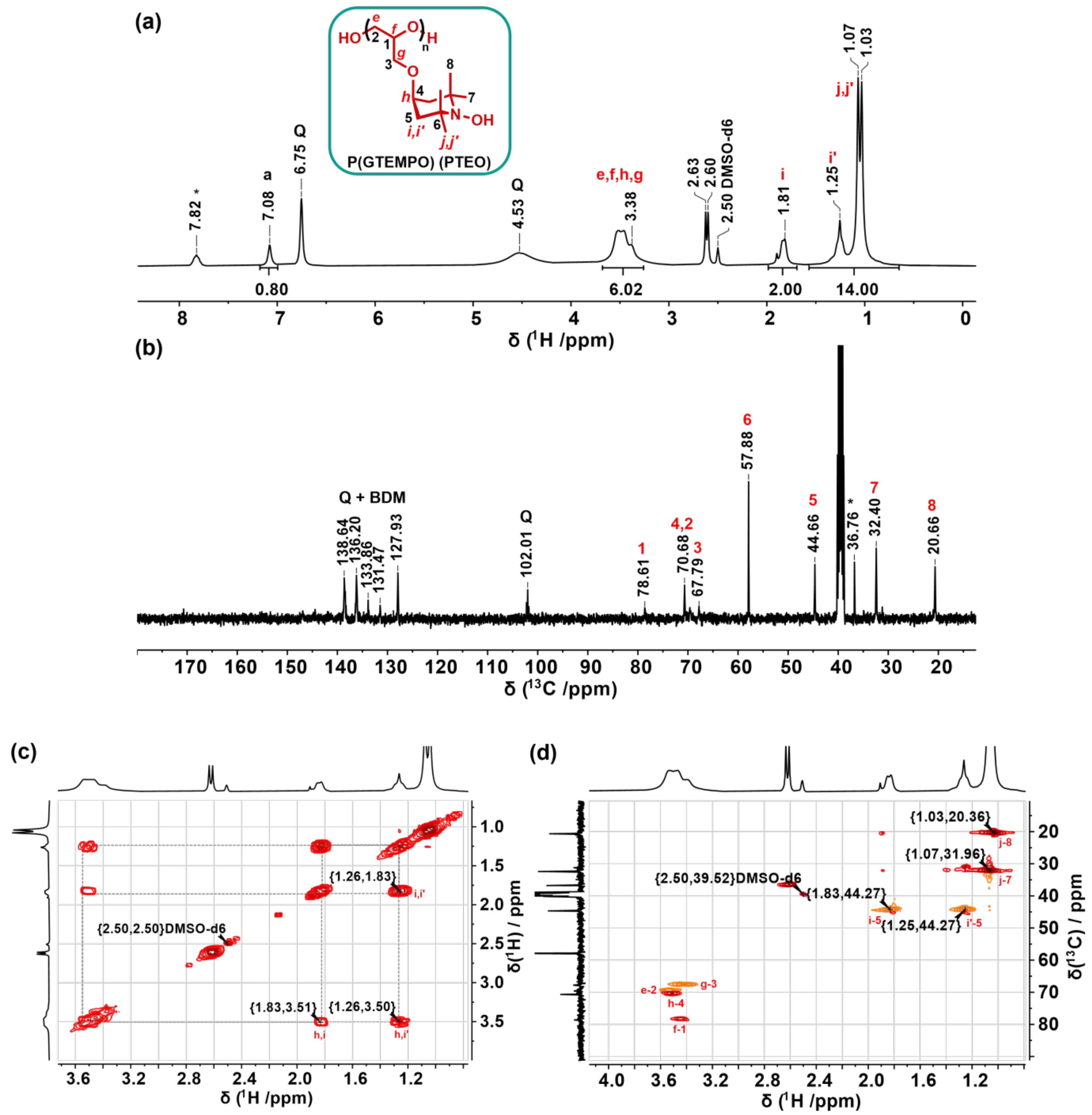

Figure S13. ^{1}H, $^{13}C\{^{1}H\}$, 2D NMR spectra (DMSO-d_6) for P(GTEMPO) (i.e. PTEO). (a) ^{1}H NMR; Q = quenching agent; *7.82 ppm impurity. (b) $^{13}C\{^{1}H\}$ NMR. *Residual DMSO. (c) COSY. (d) HSQC.

Hydrolytic Degradation Studies

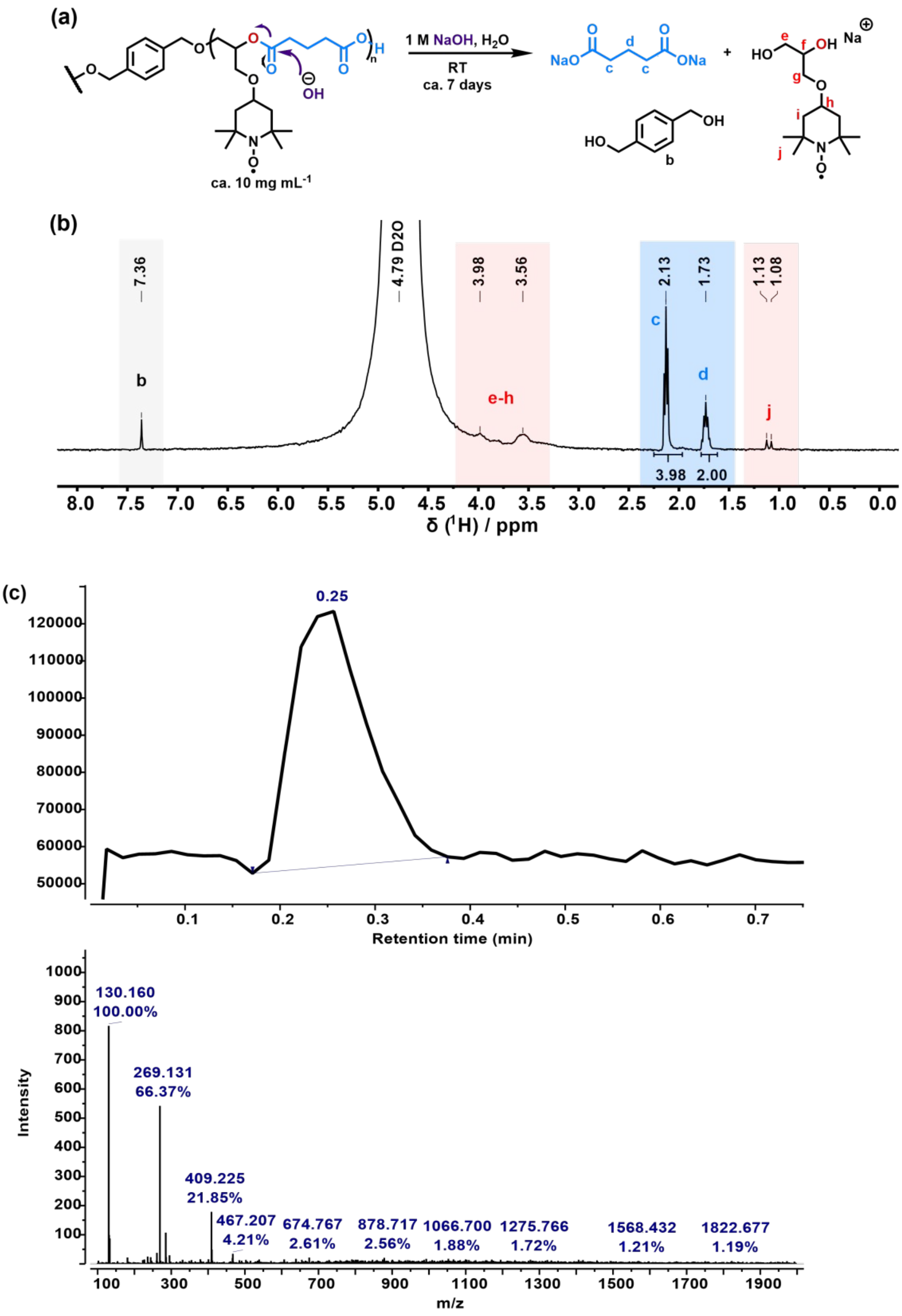

Figure S14. Proof of principle degradation for radical polyesters. (a) Reaction scheme for accelerated hydrolytic degradation of P(GTEMPO-*alt*-GA) in 1M $NaOH_{(aq)}$, which was conducted at 60 °C (<19 h) and RT (1 week). (b) ^{1}H NMR spectra (400 MHz, D_2O) of RT degradation products after ~7 days showing sodium salt of glutaric acid and GTEMPO environments. (c) Electrospray ionisation mass spectrometry (ESI-MS, positive mode) of crude degradation mixture at 60 °C supporting GTEMPO diol formation *m/z* observed = 269.13 Da; *m/z* expected for $[C_{12}H_{24}NO_4^{\bullet} + Na^{+}]$ = 269.16 Da.

Density Functional Theory (DFT)

All calculations were performed using Gaussian 16 (Rev. C.01).[3] Geometry optimisations were carried out at the ωB97X-D/6-311G(d,p) level of theory[4] at 298 K using the conductor-like polarizable continuum model (CPCM) implicit solvent model (self-consistent reaction field, SCRF approach) with a modified dielectric constant to mimic the epoxide monomer environment (solvent = THF, ε = 16, ε_{inf} = 1.867). Frequency calculations were performed at the same level of theory to confirm optimized structures as true minima (no imaginary frequencies). Calculations were performed on DP = 2 open-shell singlet diradical oligomer models representative of each P(GTEMPO-*alt*-anhydride) structure, considering head-to-tail connectivity and opposing stereocentre configurations. Natural bond orbital (NBO) analyses were performed using the pop=NBO keyword. Spin density distributions and singly occupied molecular orbital (SOMO) visualisations were obtained from formatted checkpoint (.fchk) files and rendered using GaussView (v6.0.16).[5] All DFT output files are available at https://github.com/GeorgeLGregory/Radical-Polyesters-ROCOP-Spacer-Conductivity.

Table S2. Optimised Geometries for N⋯N spacing and SOMO energy levels.

Polymer	*G (Hartree)*[a]	Spacing N⋯N (Å)[b]			SOMO (eV)[c]		
		Neutral Diradical	$TEMPO^+$/ TEMPO	$TEMPO^+$/ $TEMPO^+$	1	2	Average
GA	-2418.046154	20.0	20.1	19.9	-7.63	-7.64	-7.63
DGA	-2488.846598	19.8	19.9	20.1	-7.61	-7.63	-7.62
TDGA	-3134.842678	19.7	19.8	20.0	-7.61	-7.63	-7.62
PA	-2643.225565	7.80	7.85	8.23	-7.58	-7.62	-7.60
MPA	-2721.797979	12.2	6.00*	12.3	-7.57	-7.63	-7.60
TCA	-3117.025901	6.75	6.88	7.01	-7.62	-7.63	-7.62
HHPA	-2650.382459	11.7	6.72*	12.6	-7.63	-7.63	-7.63

[a] Gibbs free energy minimum for the TEMPO•/TEMPO• species (DP=2). [b] N⋯N distances measured from optimised geometries in GaussView. Columns 1 and 2 refer to N⋯N distances in the neutral diradical (TEMPO•/TEMPO•), singly oxidised ($TEMPO^+$/TEMPO•), and doubly oxidised ($TEMPO^+$/$TEMPO^+$) states respectively; average of columns 1 and 2 is given. *Reduced N⋯N distance in the singly oxidised state reflects geometric relaxation upon oxidation. [c] SOMO energy levels obtained from .cube files generated from formatted checkpoint (.fchk) files in GaussView; values 1 and 2 refer to each nitroxide radical centre in the DP = 2 oligomer. For comparison, the SOMO energy levels computed for poly(GTEMPO) (PTEO, DP = 2) at the same level of theory were −7.62 eV (N⋯N spacing 14.5 Å).

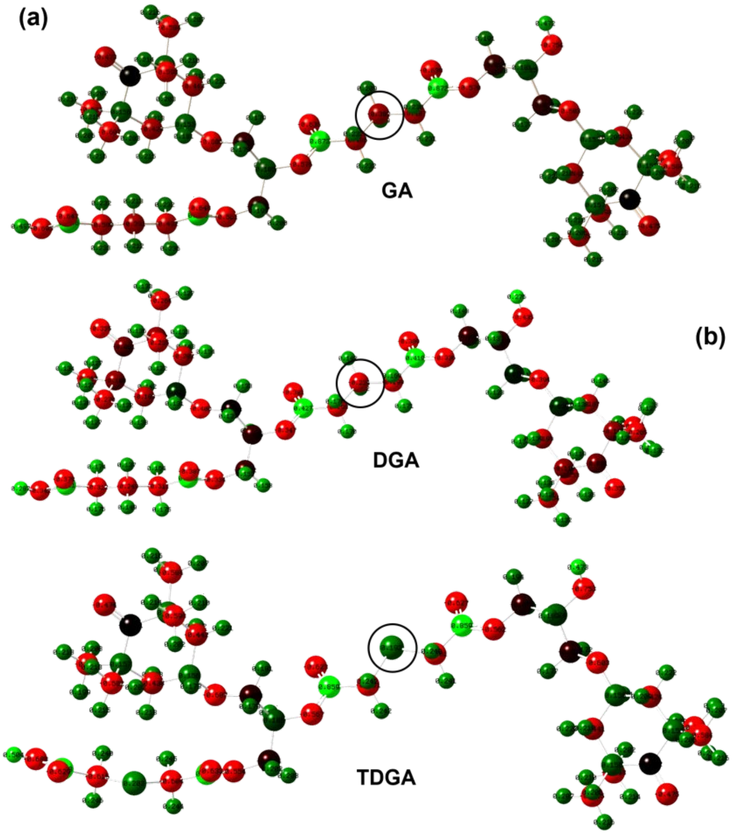

(b)

Polymer	Atom	NBO Charge (e)
GA	C (bridging)	-0.382
DGA	O (ether)	-0.561
TDGA	S	+0.203

Figure S15. Natural Bond Orbital (NBO) charges on bridging atom for isostructural flexible anhydride backbones. (a)-(b) The positive charge on sulfur in TDGA relative to the electronegative bridging atoms in GA and DGA is proposed to quench radical character via inductive withdrawal, consistent with the reduced radical content observed experimentally.

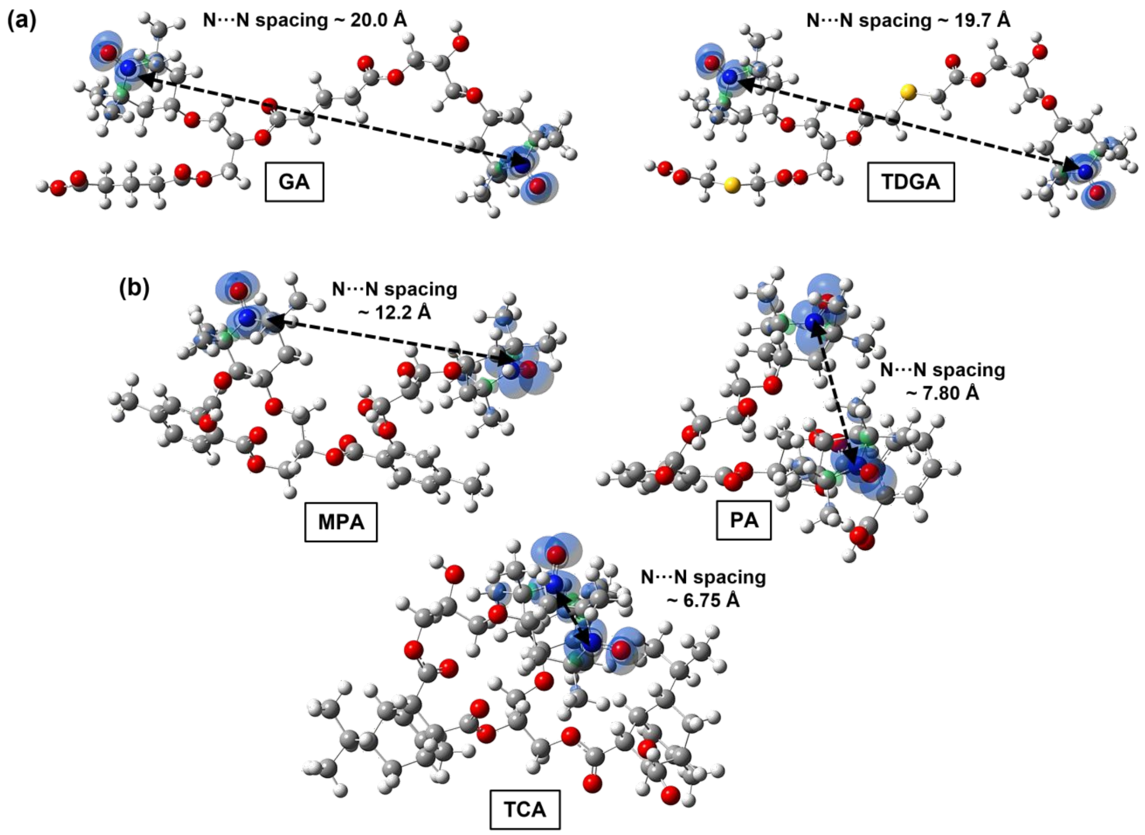

Figure S16. Spin density surfaces (isovalue = 0.002) for DFT optimised geometries. (a) Flexible spacers and (b) rigid spacers. DGA and HHPA are provided in Figure 1 of the manuscript.

Thermogravimetric Analysis (TGA)

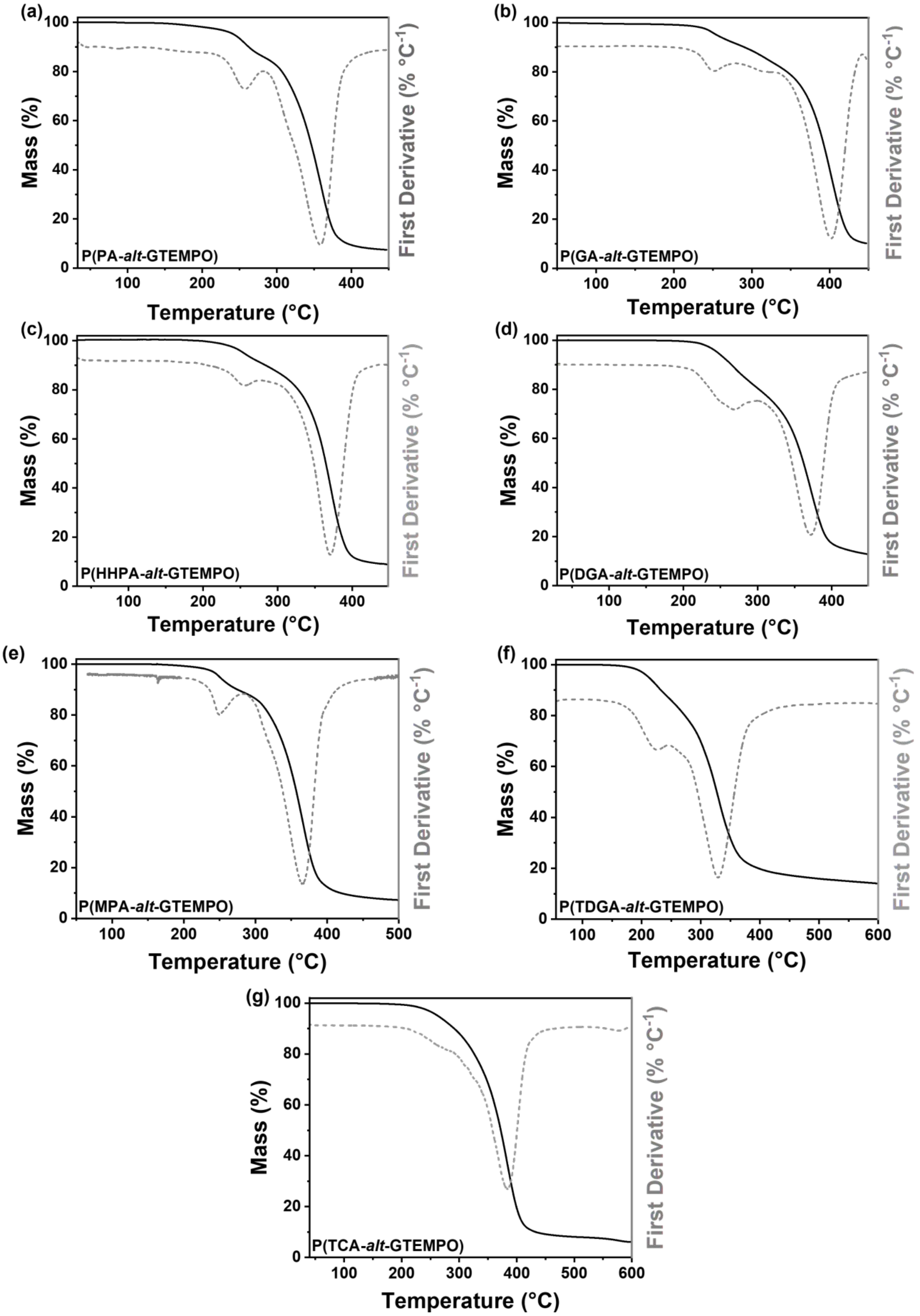

Figure S17. (a)-(g) TGA and derivative (DTG) curves for radical polyesters (Figure 2b). DTG curves were processed using Trios software (TA Instruments) smoothing function. Two degradation steps are observed: an initial mass loss of ~ 20% with DTG peak temperatures in the range 257–269 °C, tentatively attributed to degradation of the nitroxide-containing pendant group, followed by degradation of the polyester backbone at higher temperature.

SQUID Magnetometry – Curie-Weiss Analysis

Molar susceptibility (χ, emu Oe^{-1} mol^{-1}) was calculated from the measured magnetic moment according to Equation S3:

$$\chi = \frac{M}{H} \times \frac{M_r}{m} \quad \textbf{Equation S3}$$

where *M* is the magnetic moment at a given temperature (emu), *H* is the applied magnetic field (Oe), M_r is the repeat unit molar mass weighted for the BDM initiating group (g mol^{-1}), and *m* is the sample mass (g). A constant diamagnetic correction (χ_{dia}, Table S3) was applied to χ for each polymer, calculated from Pascal's constants summed over the repeat unit:[6]

$$\chi_{corrected} = \chi_{measured} - \chi_{dia}$$

Samples were measured in a Quantum design MPMS-3 Magnetometer, held within a gelatine capsule inside in a plastic straw. Background subtraction was performed in SquidLab (v2.9.3)[7] using a separate measurement of an empty gelatine capsule and straw under identical conditions (1 kOe, 1.8–313 K). Background-subtracted scans were fitted to extract the magnetic moment at each temperature. Variable-temperature susceptibility measurements were performed on all samples under field-cooled (FC) conditions. For a representative flexible and rigid spacer, zero-field cooled (ZFC) measurements were also collected; ZFC and FC plots were coincident in both cases, confirming the absence of magnetic ordering or spin-glass behaviour (Figure S18). FC data were used for Curie-Weiss analysis throughout.

Inverse molar susceptibility (χ^{-1}) was plotted as a function of temperature (*T*) in accordance with the Curie-Weiss law. A linear fit was applied over the temperature regime exhibiting ideal Curie-Weiss behaviour (see Table S3 footnote) to extract the Curie constant *C*:

$$X^{-1} = \frac{T}{C} - \frac{\theta_{CW}}{C} \quad \textbf{Equation S4}$$

where θ_{CW} is the Weiss constant. The effective magnetic moment (μ_{eff}) was calculated from *C* according to:

$$\mu_{eff} = \sqrt{\frac{3k_B C}{\mu_B^2 N_A}}\mu_B \approx \sqrt{8C}\,\mu_B \quad \textbf{Equation S5}$$

The radical content per repeat unit was determined as the ratio of the observed μ_{eff} to that expected for a spin-½ species with a single unpaired electron:

$$\mu_{calc} = \sqrt{S(S+1)}\mu_B = \sqrt{3}\,\mu_B \quad \textbf{Equation S6}$$

$$\textit{Radical content per repeat unit} = \frac{\mu_{eff}}{\mu_{calc}} \approx \frac{\sqrt{8C}}{\sqrt{3}} \quad \textbf{Equation S7}$$

Some non-linearity can be observed in χ^{-1} vs T. For comparison, *C* was also extracted by direct nonlinear least-squares regression (NLS) of χ vs T to the Curie-Weiss law using the Levenberg–Marquardt algorithm in OriginPro 2026, with *C* and θ_{CW} as free parameters. A comparison of Curie constant obtained using a linear fit to the high temperature region of χ^{-1}

vs T, and a nonlinear least-squares regression (NLS) fit of χ vs T for the entire region shows minimal impact on the extracted radical content per repeat unit (Table S3). θ_{CW} values obtained by nonlinear regression are reported throughout; all values are small and negative, indicative of weak antiferromagnetic exchange interactions between radical centres.

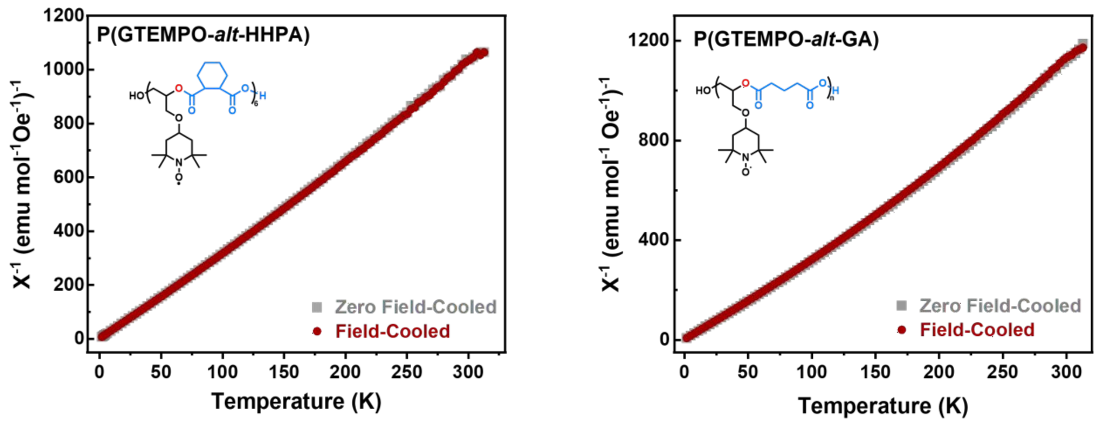

Figure S18. Zero-field cooled (ZFC) and field-cooled (FC) Curie-Weiss curves were coincident for representative rigid and flexible spacers measured under both conditions (GA, HHPA); FC data were therefore used throughout. Data shown prior to background subtraction and diamagnetic correction.

Table S3. SQUID magnetometry data for radical content determination (Figure 3b, S19).[a]

Polymer	*m* (mg)	$M_{r,\ repeat\ unit}$ (g mol^{-1})	$M_{r,\ weighted}$ (g mol^{-1})[b]	χ_{dia} (×10^{-4} emu mol^{-1} Oe^{-1})	*C* (emu K mol^{-1})[c]		θ_{CW} (K)[d]	Radical Content (%)[e]	
					Linear	NLS		Linear	NLS
PA	8.1	376.4	391.1	-2.3	0.344	0.365	-0.574	98	98
HHPA	5.0	382.5	406.7	-2.5	0.314	0.285	-0.456	91	87
MPA	14.6	390.5	407.0	-2.4	0.384	0.359	-0.544	99	98
TCA	13.6	462.6	496.9	-3.1	0.375	0.360	-0.448	99	98
GA	12.5	342.4	360.1	-2.2	0.341	0.335	-0.575	95	95
DGA	5.5	344.4	371.8	-2.1	0.270	0.280	-0.456	85	86
TDGA	6.3	360.4	383.7	-2.2	0.114	0.111	-0.185	55	54

[a] Applied magnetic field (H) = 1 kOe. [b] $M_{r,\ weighted} = \frac{M_{n,SEC}}{n}$; $n = \frac{M_{n,\ SEC} - M_{r,\ BDM}}{M_{r,\ repeat\ unit}}$ where $M_{r,BDM}$ = 138.16 g mol^{-1}, $M_{n,SEC}$ and n values are provided in Scheme 1 of the manuscript. [c] Curie Constant from linear fit of χ^{-1} vs T ($\chi^{-1} = \frac{T}{C} - \frac{\theta_{CW}}{C}$ Equation S4) over T range at which the 'DC free fit' is ≥ 0.9 or nonlinear least squares regression (NLS) of χ vs T over full T range (1.8 to 312 K); [d] From nonlinear regression of χ vs T. [e] Calculated from *C* using $\text{Radical content}\ \boldsymbol{per\ repeat\ unit} = \frac{\mu_{eff}}{\mu_{calc}} \approx \frac{\sqrt{8C}}{\sqrt{3}}$ **Equation S7**.

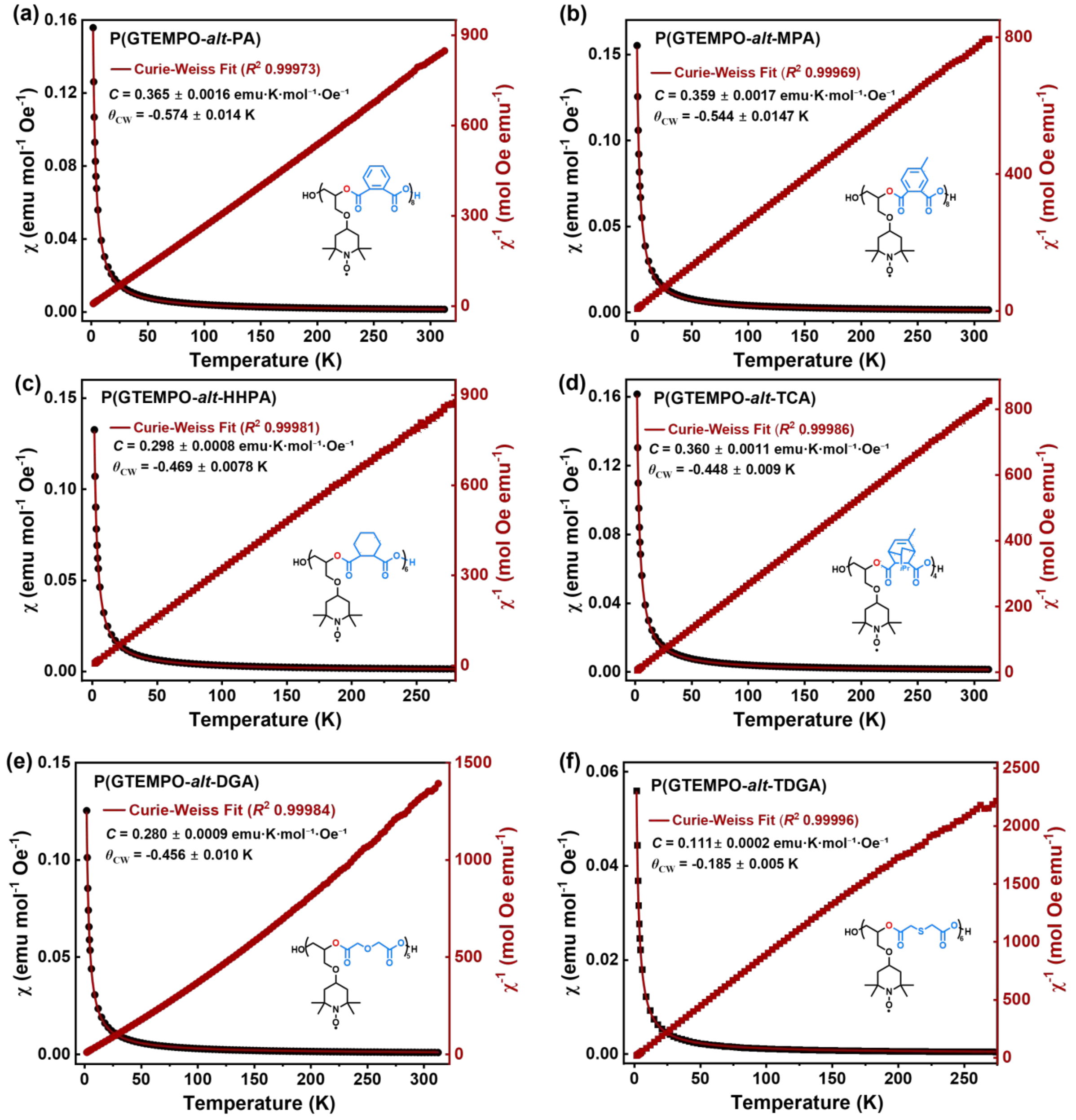

Figure S19. χ and χ^{-1} as a function of T for P(GTEMPO-*alt*-anhydrides), with Curie-Weiss fits used to determine *C* for calculation of radical content. Data shown after background subtraction and diamagnetic correction. The plot for P(GTEMPO-*alt*-GA) is provided in Figure 3a of the manuscript.

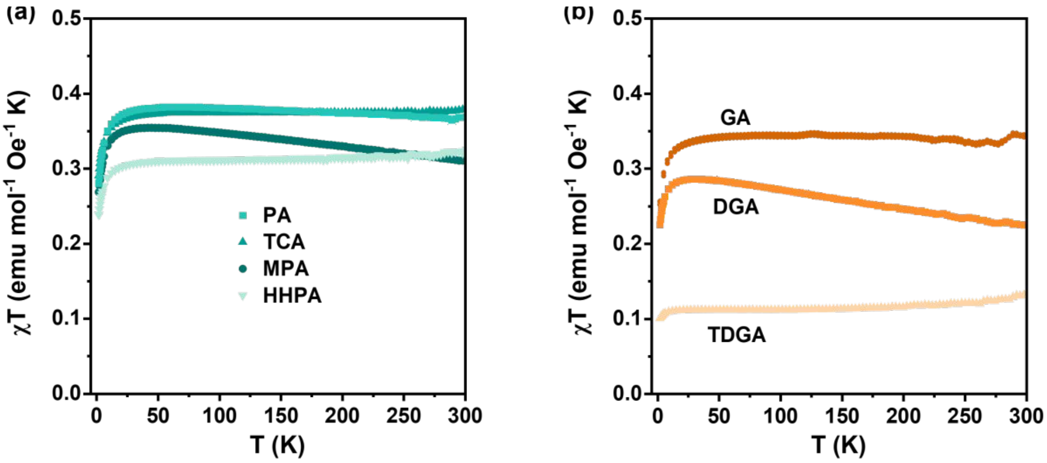

Figure S20. χT vs T for poly(GTEMPO-*alt*-anhydrides): (a) rigid and (b) flexible spacers. χT drops below ~26-30 K reflecting the weak antiferromagnetic exchange indicated by the Curie-Weiss fits (Figure 3a, Table S3). Above this, χT is approximately constant, consistent with paramagnetic behaviour. A gradual decline of ~13% (MPA) and ~22% (DGA), observed across 30-300 K is attributed to small residual diamagnetic background signals.

Electron Paramagnetic Resonance (EPR) Spectroscopy

Polymer solutions were prepared in toluene at known concentration. The theoretical spin concentration ($[\text{Spins}]_{\text{theory}}$) was calculated from the solution concentration assuming 100% active TEMPO radicals per repeat unit:

$$[\mathbf{Spins}]_{\mathbf{theory}} = \frac{c \times 10^6}{M_{r,weighted}} \quad \textbf{Equation S8}$$

where c is the mass concentration of the polymer solution (mg mL⁻¹) and $M_{r,\text{weighted}}$ the repeat unit molar mass weighted for the BDM initiating group (g mol⁻¹, Table S3), assuming one active TEMPO radical per repeat unit (100% radical content). The factor 10^6 converts from mol L⁻¹ to μM.

EPR measurements were performed at room temperature on P(GTEMPO-*alt*-GA) and P(GTEMPO-*alt*-PA) as representatives of the flexible and rigid anhydride spacer series, respectively. The number of spins per unit volume (spins mm⁻³) was determined by double integration of the EPR absorption spectrum, and the total spin count converted to an observed solution concentration ($[\text{Spins}]_{\text{obs}}$) for direct comparison with $[\text{Spins}]_{\text{theory}}$.

$$[\mathbf{Spins}]_{\mathbf{obs}} = \frac{N_{obs}}{(N_A \times V) \times 10^6} \quad \textbf{Equation S9}$$

where N_{obs} is the total spin count from double integration (spins), N_A is Avogadro's number (mol⁻¹), and V is the sample volume (L).

Table S4. Comparison of theoretical and observed spin concentrations by EPR.

P(anhydride-*alt*-GTEMPO)	Spins mm^{-3}	Spins	$[\text{Spins}]_{\text{theory}}$ (μM)[a]	$[\text{Spins}]_{\text{obs}}$ (μM)
PA	5.162×10^{13}	1.460×10^{16}	107	86
GA	4.078×10^{13}	1.005×10^{16}	93	68

FT-IR Spectrum

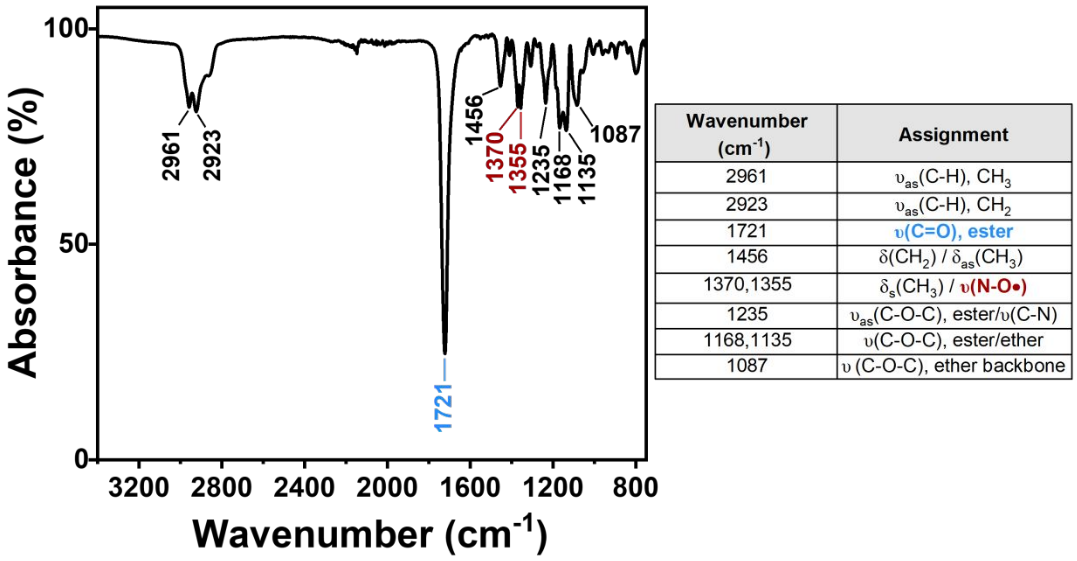

Wavenumber (cm^{-1})	Assignment
2961	υ_{as}(C-H), CH_3
2923	υ_{as}(C-H), CH_2
1721	υ(C=O), ester
1456	$\delta(CH_2)$ / $\delta_{as}(CH_3)$
1370,1355	$\delta_s(CH_3)$ / υ(N-O•)
1235	υ_{as}(C-O-C), ester/υ(C-N)
1168,1135	υ(C-O-C), ester/ether
1087	υ (C-O-C), ether backbone

Figure S21. ATR-FTIR spectra for P(GTEMPO-*alt*-GA) (baseline corrected). Key absorptions are labelled. Band assignments: v = stretch, δ = deformation, *as* = asymmetric, *s* = symmetric. The absence of bands characteristic of hydroxylamine (N–OH, ~3200 cm^{-1}) and oxoammonium (N=O, ~1540 cm^{-1}) confirms retention of the nitroxide radical functionality and high radical content per repeat unit quantified by EPR and SQUID.

Cyclic Voltammetry (CV)

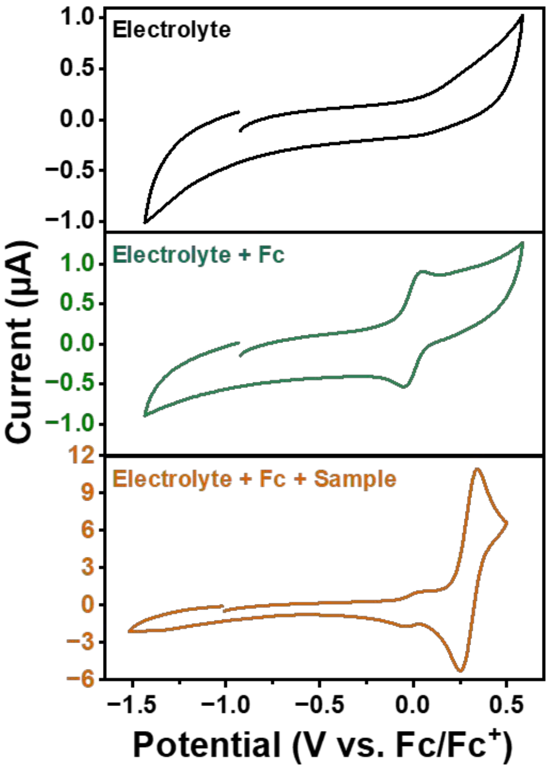

Figure S22. CVs (ca. 0.1 M [*t*-Bu_4N][PF_6] in MeCN), 0.1 V s^{-1}: electrolyte background (black), electrolyte with ferrocene (Fc) (green), and poly(GTEMPO-*alt*-GA) (ca. 5 mM) with Fc internal reference (orange). Potentials are referenced to the Fc/Fc$^+$ couple, each Fc-containing trace being referenced to its own internal Fc. The poly(GTEMPO-*alt*-GA) couple at $E_{1/2} \approx$ 0.30 V vs Fc/Fc$^+$ is consistent with the TEMPO•/TEMPO$^+$ oxidation (enlarged in Figure 3c).

Solid-State Electrical Conductivity

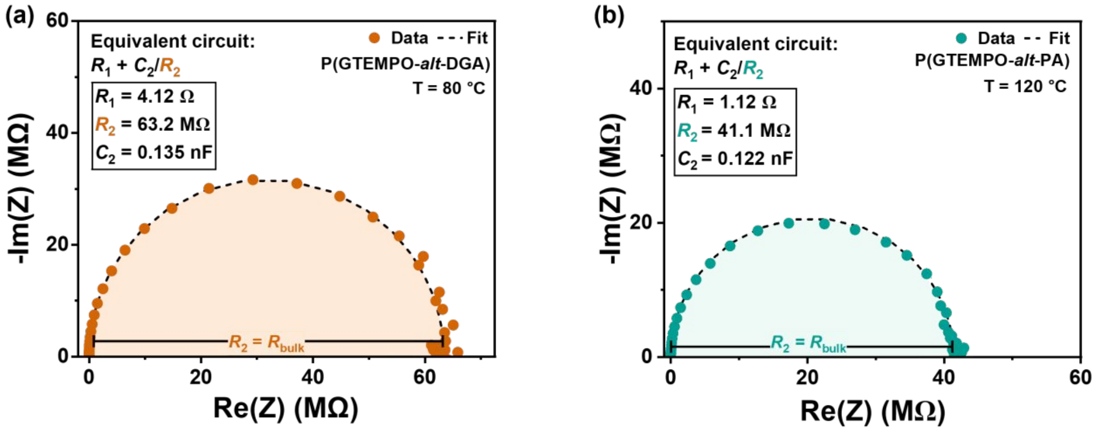

Figure S23. Nyquist plots from EIS and equivalent-circuit fits for rigid and flexible spacers supporting a bulk-dominated transport response. The data are well described by a single semicircle fitted to a series resistance (R_1) combined with a parallel resistor (R_2) and capacitor (C_2). The picofarad-scale capacitance (e.g., $C_2 \approx 140$ pF for DGA), characteristic of a bulk geometric response rather than a microfarad-scale electrode/interfacial process, identifies R_2 as the bulk resistance (R_{bulk}, 41-63 MΩ) and agrees with that obtained from the DC measurements. The series resistance (R_1, 1-4 Ω) is ca. 7 orders of magnitude smaller than R_{bulk} and reflects negligible wire resistances. The semicircle fully returns to the real axis at low frequency (higher Re(Z)), with no low-frequency tail or additional arc, further indicating good electrode contact and no additional interfacial regions. The absence of a low-frequency Warburg feature is consistent with electronic, rather than ion-diffusion-limited, transport.

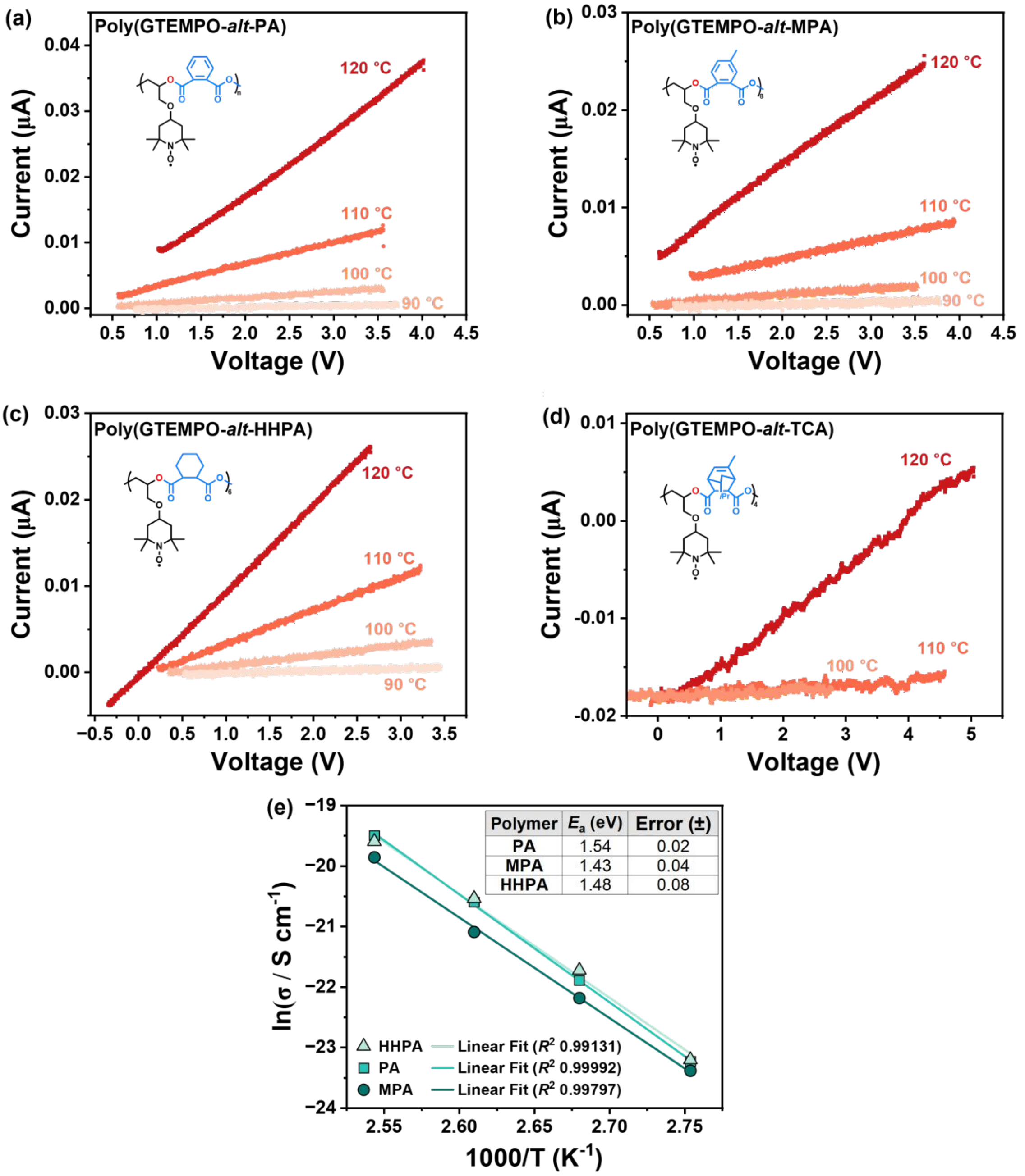

Figure S24. I-V curves (representative of 3 repeats) from LSV (Au|polyester|Au; A = 0.317 cm^2) at temperatures above T_g for rigid spacers. (a) PA (L = 0.109 cm). (b) MPA (L = 0.115 cm). (c) HHPA (L = 0.098 cm). (d) TCA (L = 0.110 cm). The slope of curves (=1/R) at 120 °C were used to determine σ in Figure 4b using $\boldsymbol{\sigma} = \frac{\quad}{\mathbf{LA \times R}}$ **Equation** S1. (e) Arrhenius plots of ln(σ) vs 1/T. Activation energies (E_a) extracted from the slopes are provided in the table (inset).

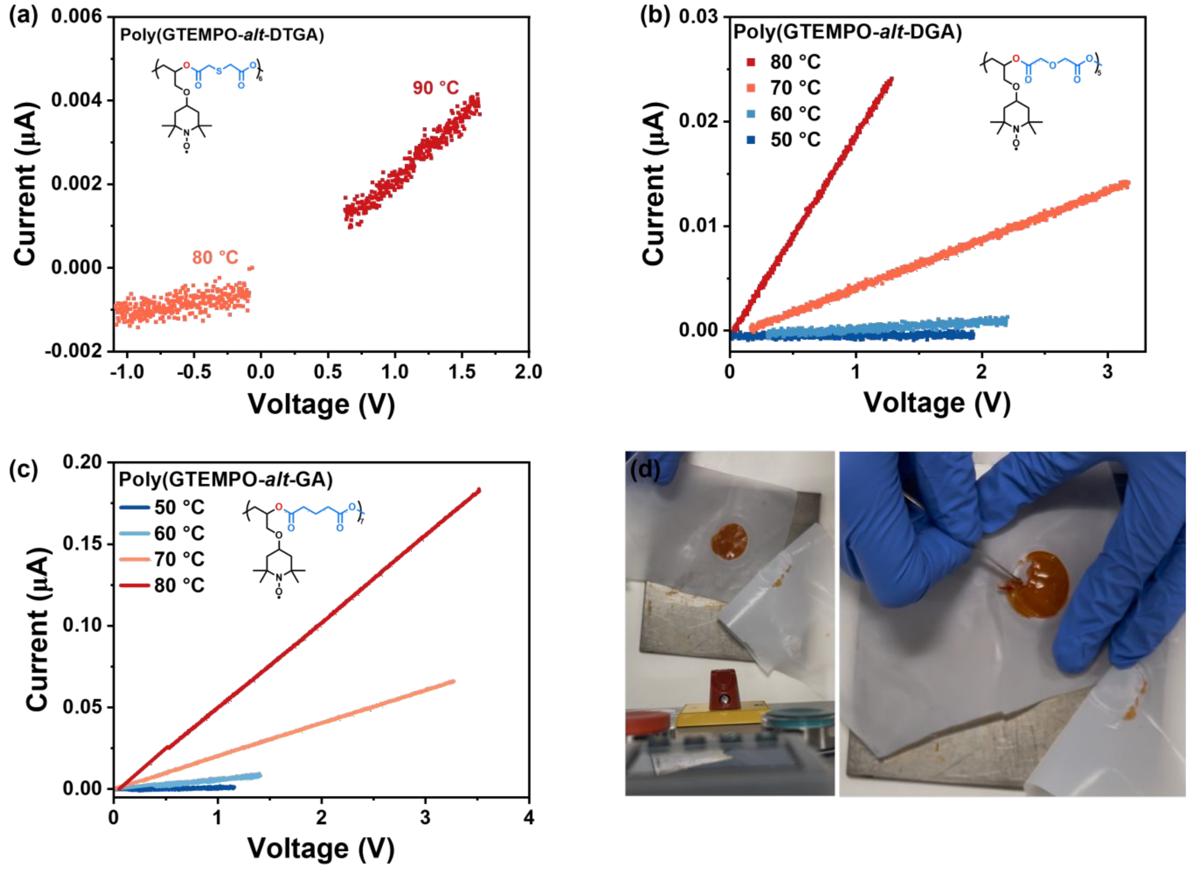

Figure S25. I-V curves from LSV (Au|polyester|Au; A = 0.317 cm^2) at temperatures above T_g for flexible spacers (L = 0.09 cm). (a) TDGA. (b) DGA. (c) GA. The slope of curves (=1/R) at 80 °C were used to determine σ in Figure 4c using $\boldsymbol{\sigma} = \frac{\quad}{\mathbf{LA \times R}}$ **Equation** S1. (d) Hot-pressed film of poly(GTEMPO-*alt*-GA).

Blend Study

Preparation Procedure: Blends of poly(GTEMPO-*alt*-GA) and poly(GTEMPO-*alt*-PA) were prepared at GA:PA ratios of 90:10, 80:20, and 70:30 (w/w) by solution blending. For each blend, for example, for the 90:10 (w/w) blend, poly(GTEMPO-*alt*-GA) (100 mg) was dissolved in anhydrous DCM (1 mL) and added to a solution of poly(GTEMPO-*alt*-PA) (11 mg) in DCM (ca. 0.4 mL). The combined solution was stirred overnight at room temperature before being poured into a Teflon mould and left to air dry under ambient conditions for 48 h. The blend film was subsequently dried under vacuum at 60 °C (2.3 × 10^{-2} mbar) for at least 48 h until constant mass and no residual DCM was observed by ^{1}H NMR spectroscopy.

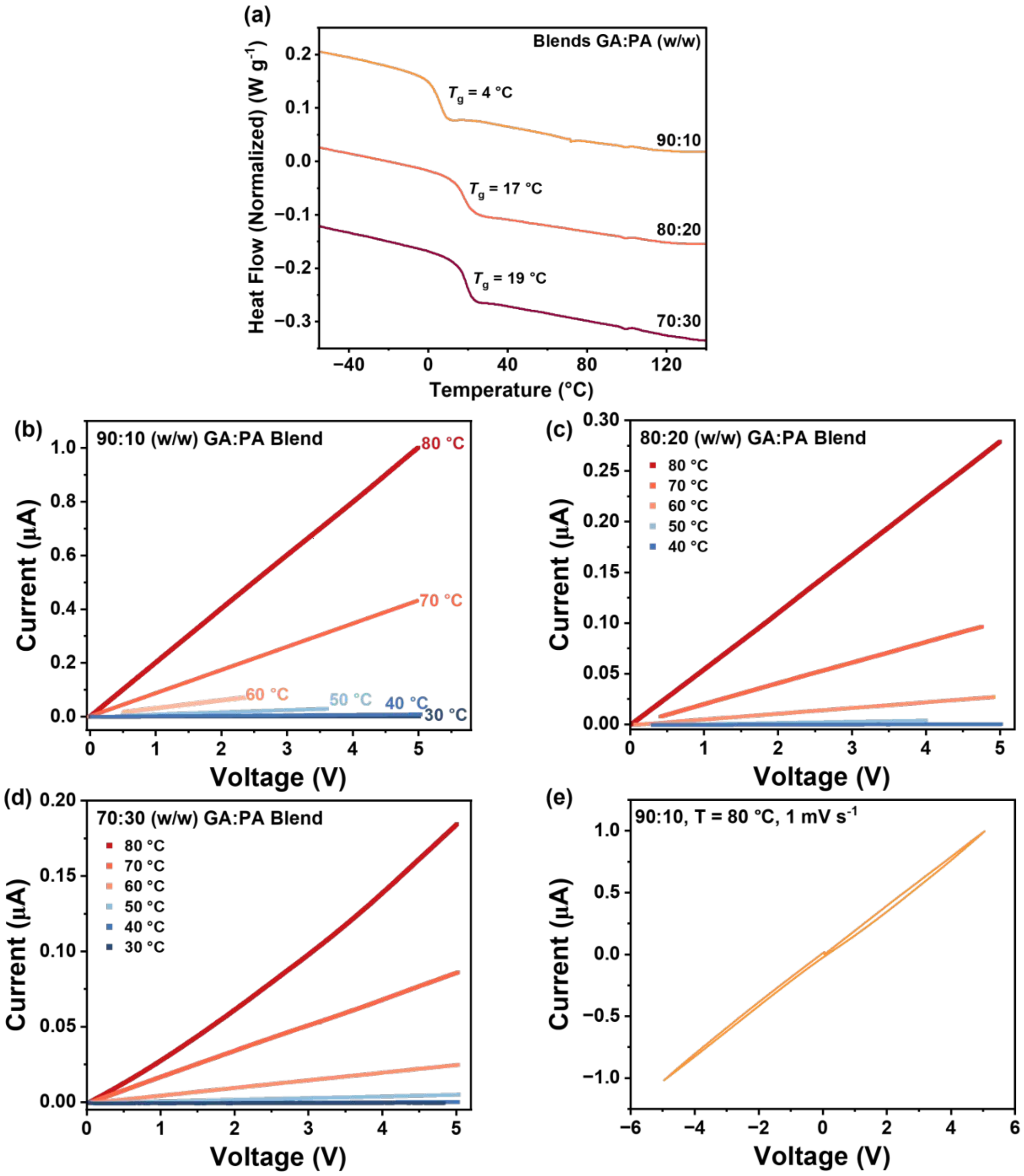

Figure S26. Characterisation of P(GTEMPO-*alt*-GA)/P(GTEMPO-*alt*-PA) blends. (a) Stacked DSC traces 90:10, 80:20, and 70:30 (w/w) blends (third heating cycle, 10 °C min^{-1}). (b)-(d) LSV at different temperatures for blend compositions. (e) CV solid-state from -5 V to + 5 V for 90:10 blend.

PCL-*b*-P(GTEMPO-*alt*-PA)-*b*-PCL

Synthetic procedure: GTEMPO (0.4 g, 8.4 mmol, 60 equiv.), PA (0.2 g, 5.6 mmol, 40 equiv.) and ε-caprolactone (εCL, 0.8 mL, 7.2 mmol, 50 equiv.) were dissolved in toluene (2 mL) in an oven-dried Schlenk flask. *t*-Bu-P_2 (50 uL, ca. 2.0 M in THF, 1 equiv.) and BDM (20 mg, 0.14 mmol, 1 equiv.) were added. The stirring solution was heated to 60 °C. GTEMPO/PA ROCOP proceeded first, with the catalyst switching to εCL ROP upon consumption of the anhydride. Reaction progression was monitored by ^{1}H NMR of periodic aliquots (quenched with benzoic or acetic acid) and M_n evolution by SEC, confirming sequential block formation (Figure S27).

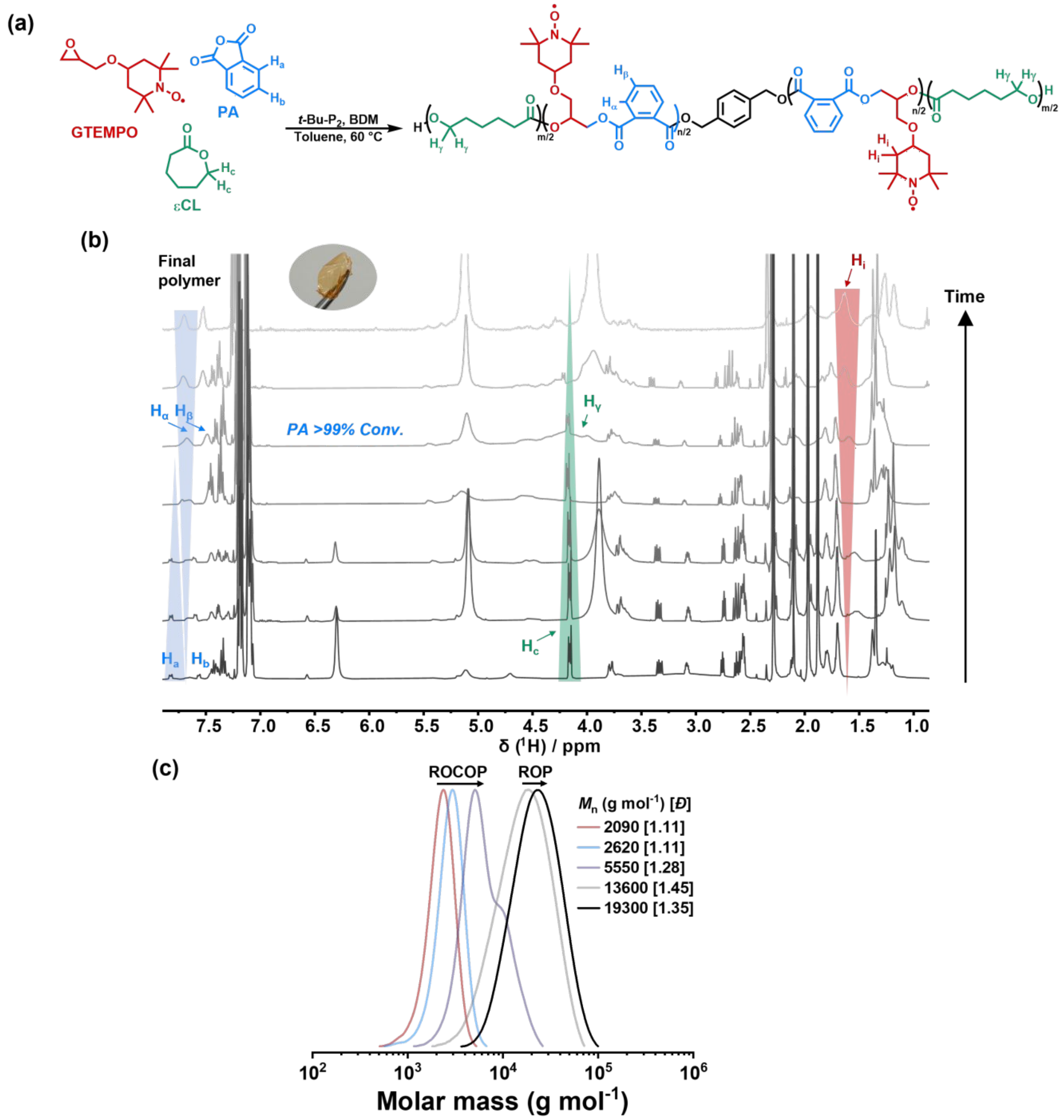

Figure S27. Synthesis and characterization of PCL-*b*-P(GTEMPO-*alt*-PA)-*b*-PCL. (a) Reaction scheme. (b) ^{1}H NMR spectra ($CDCl_3$) of aliquots showing GTEMPO/PA ROCOP followed by ε-CL ROP and (c) corresponding M_n evolution by SEC (THF eluent, relative to PS standards). Inset: photograph of a representative film. The characteristic orange colouration, present for all polymers in this work is consistent with the TEMPO nitroxide radical. Purified polymer $M_{n,\,SEC}$ = 19.9 kg mol^{-1} ($Đ$ = 1.40).

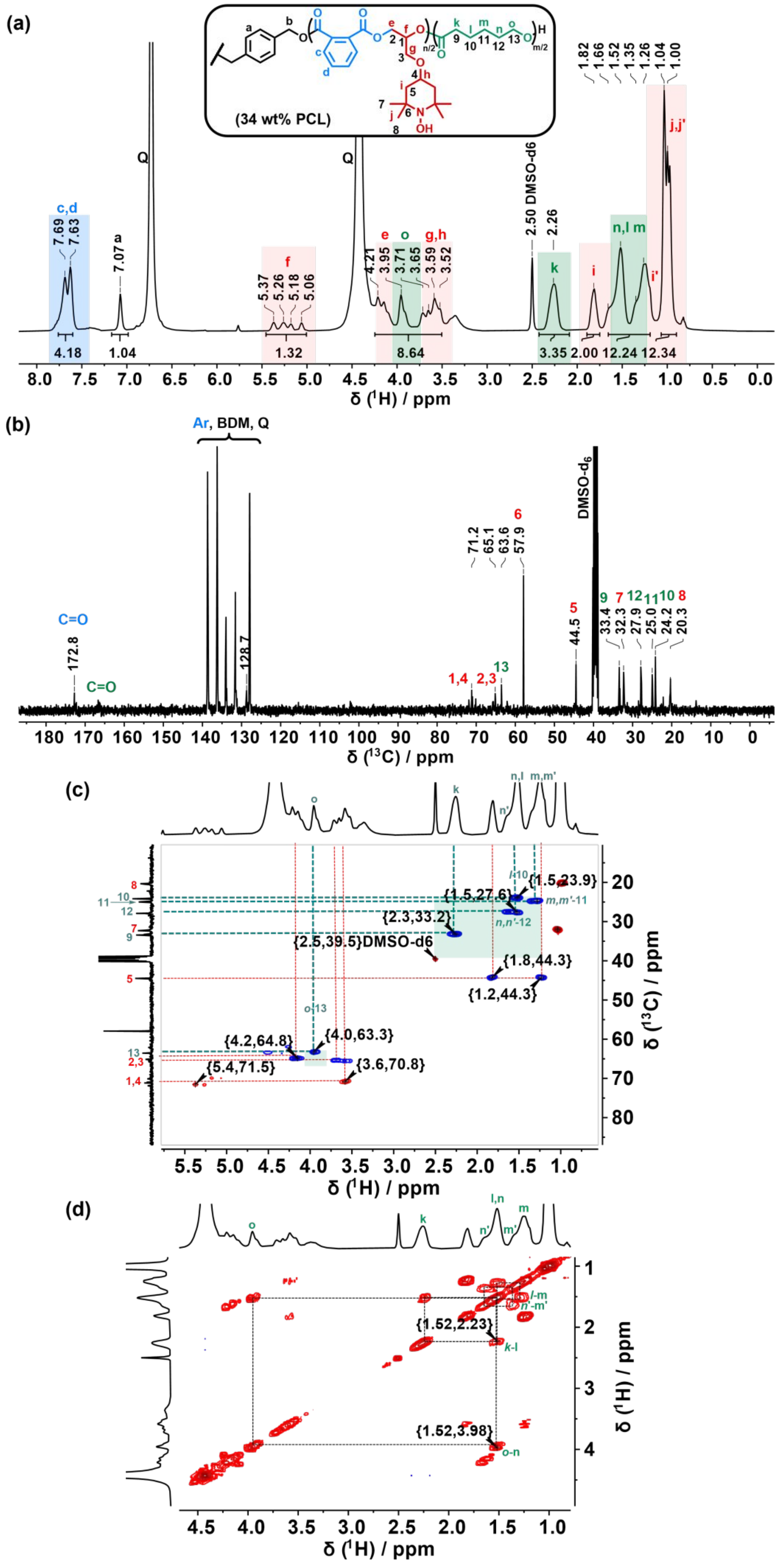

(a)
(34 wt% PCL)
c,d
7.69
7.63
a
7.07
Q
f
5.37
5.26
5.18
5.06
e
o
g,h
4.21
3.95
3.71
3.65
3.59
3.52
2.50 DMSO-d6
2.26
k
i
n,l m
i'
j,j'
1.82
1.66
1.52
1.35
1.26
1.04
1.00
4.18
1.04
1.32
8.64
3.35
2.00
12.24
12.34
δ (^{1}H) / ppm
(b)
Ar, BDM, Q
C=O
172.8
C=O
128.7
1,4
2,3
13
71.2
65.1
63.6
57.9
6
DMSO-d6
5
44.5
9
7
12
11
10
8
33.4
32.3
27.9
25.0
24.2
20.3
δ (^{13}C) / ppm
(c)
{1.5,27.6}
{1.5,23.9}
{2.3,33.2}
{2.5,39.5}DMSO-d6
{1.8,44.3}
{1.2,44.3}
{4.2,64.8}
{4.0,63.3}
{5.4,71.5}
{3.6,70.8}
l-10
m,m'-11
n,n'-12
o-13
δ (^{1}H) / ppm
δ (^{13}C) / ppm
(d)
{1.52,2.23}
{1.52,3.98}
l-m
n'-m'
k-l
o-n
δ (^{1}H) / ppm
δ (^{1}H) / ppm

(e)

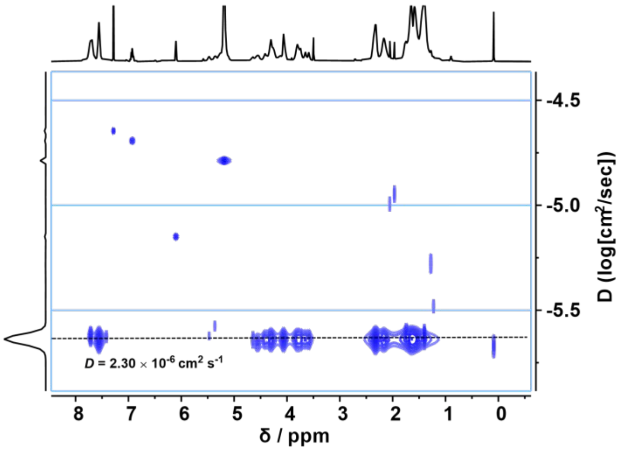

Figure S28. ^{1}H, $^{13}C\{^{1}H\}$ and 2D NMR spectra of PCL-*b*-P(GTEMPO-*alt*-PA)-*b*-PCL. (a) ^{1}H NMR spectra (DMSO-d_6); wt% PCL determined by relative integration of environment *i* at 1.82 ppm (2H, ROCOP block) and *k* at 2.26 ppm (2H, PCL block) using repeat unit masses of $M_{r,\ ROCOP}$ = 376.4 g mol^{-1} and $M_{r,\ PCL}$ = 114.14 g mol^{-1} as: ((3.35×114)/((3.35×114)+(2×376.4))) × 100 = 34 wt% PCL. (b) $^{13}C\{^{1}H\}$ NMR spectra (DMSO-d_6) and (c) HSQC showing environments at 1.55-1.62 ppm are composed of *n* and *l* PCL protons: *n, n'* correlate to the same ^{13}C environment at 33 ppm and *l* to C10 at 24 ppm. (d) COSY (DMSO-d_6); cross-peak assignments for the ROCOP block are detailed above (**Figure S7**). (e) DOSY 2D NMR ($CDCl_3$) confirming block copolymer formation over a mixture of homopolymers. Impurities are attributed to solvent and pentafluorophenylhydrazine used to quench the sample.

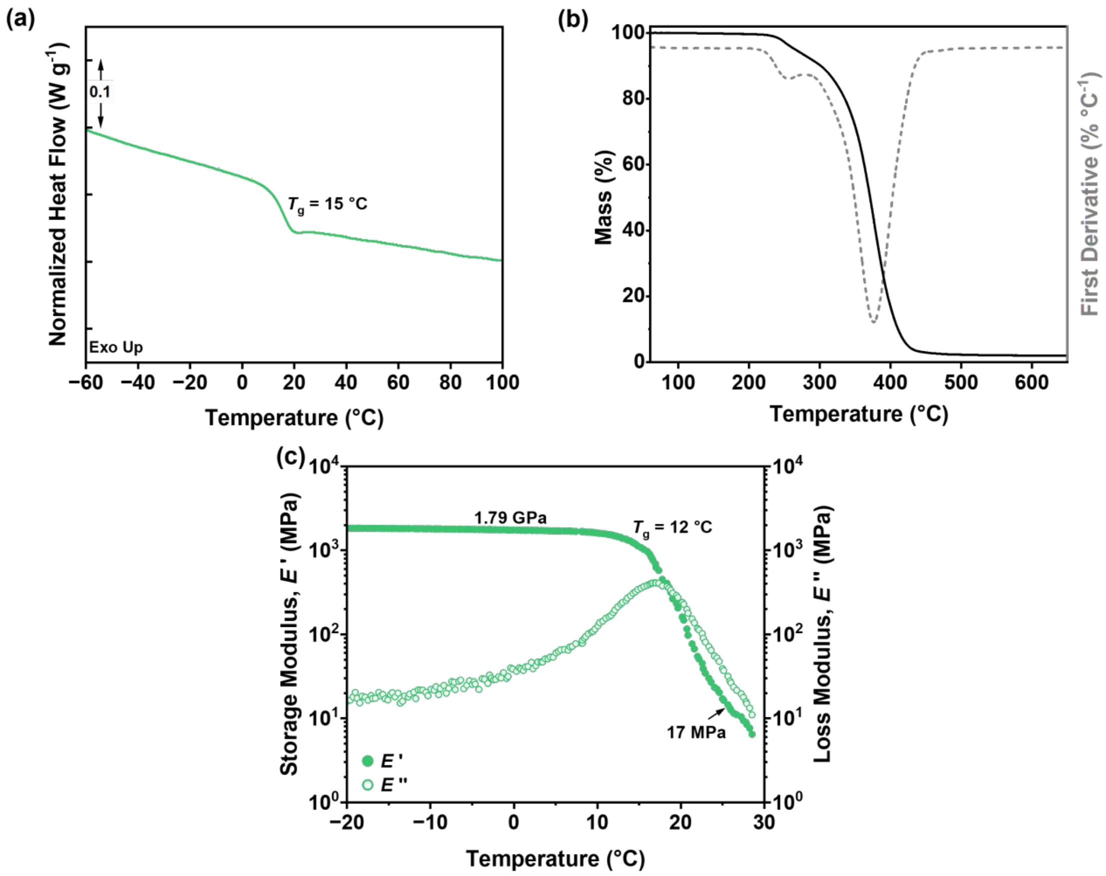

Figure S29. Thermal properties of PCL-*b*-P(GTEMPO-*alt*-PA)-*b*-PCL. (a) DSC; (b) TGA and first derivative (smoothed in TA instruments Trios software as above), $T_{d,5\%}$ = 265 °C. (c) Dynamic mechanical thermal analysis (DMTA) of standalone film under tension mode (3 °C min^{-1} heating rate, 1 Hz frequency); T_g taken as the onset of the drop in *E*' is consistent with that recorded by DSC (10 °C min^{-1} heating rate).

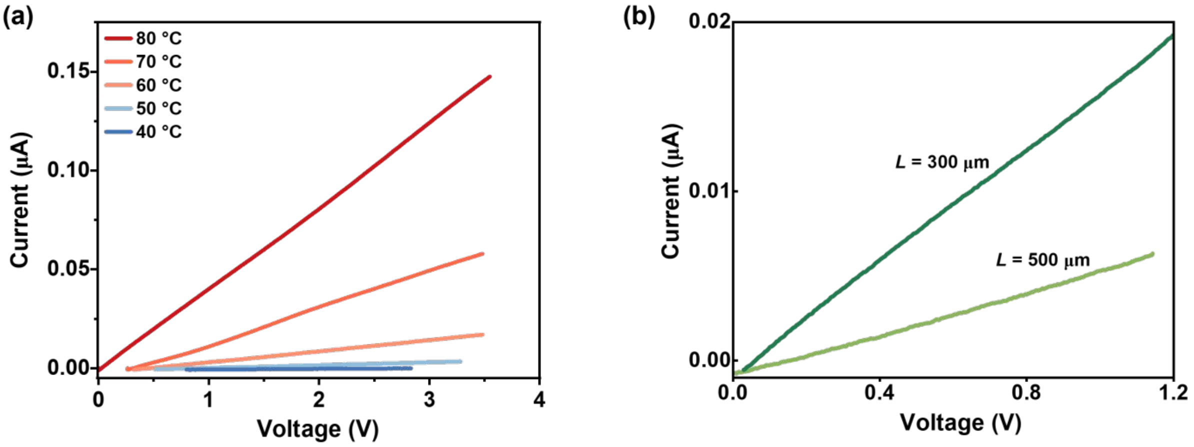

Figure S30. I-V curves for PCL-*b*-P(GTEMPO-*alt*-PA)-*b*-PCL. (a) Temperature dependence at *L* = 900 μm and (b) film thickness dependence at T = 60 °C.

References

(1) Dakshinamoorthy, D.; Weinstock, A. K.; Damodaran, K.; Iwig, D. F.; Mathers, R. T. Diglycerol-Based Polyesters: Melt Polymerization with Hydrophobic Anhydrides. *ChemSusChem* **2014**, *7* (10), 2923–2929.

(2) Jia, Z.; Fu, Q.; Huang, J. Synthesis of poly(ethylene oxide) with pending 2,2,6,6-tetramethylpiperidine-1-oxyl groups and its further initiation of the grafting polymerization of styrene. *J. Polym. Sci. Part A: Polym. Chem.* **2006**, *44* (12), 3836–3842.

(3) Frisch, M. J.; Trucks, G. W.; Schlegel, H. B.; Scuseria, G. E.; Robb, M. A.; Cheeseman, J. R.; Scalmani, G.; Barone, V.; Petersson, G. A.; Nakatsuji, H.; et al. *Gaussian 16 Rev. C.01*; Gaussian, Inc., Wallingford CT, **2016**.

(4) Chai, J.-D.; Head-Gordon, M. Long-range corrected hybrid density functionals with damped atom–atom dispersion corrections. *Phys. Chem. Chem. Phys.* **2008**, *10* (44), 6615–6620.

(5) Roy Dennington; Todd A. Keith; John M. Millam. *GaussView, Version 6*; Semichem Inc. Shawnee Mission KS, **2016**.

(6) Bain, G. A.; Berry, J. F. Diamagnetic Corrections and Pascal's Constants. *J. Chem. Educ.* **2008**, *85* (4), 532.

(7) Coak, M. J.; Jarvis, D.; Cheng, L. *SquidLab*; University of Warwick, Department of Physics ; University of Cambridge: Coventry, UK ; Cambridge, UK, **2019**. https://wrap.warwick.ac.uk/id/eprint/129665/.
